# Transgelin is a TGF*β*-inducible gene that regulates osteoblastic and adipogenic differentiation of human skeletal stem cells through actin cytoskeleston organization

**DOI:** 10.1038/cddis.2016.196

**Published:** 2016-08-04

**Authors:** M Elsafadi, M Manikandan, R A Dawud, N M Alajez, R Hamam, M Alfayez, M Kassem, A Aldahmash, A Mahmood

**Affiliations:** 1 Stem Cells Unit, Department of Anatomy, College of Medicine, King Saud University, Riyadh 11461, Kingdom of Saudi Arabia; 2 KMEB, Department of Endocrinology, University Hospital of Odense and University of Southern Denmark, Odense, Denmark; 3 Berlin-Brandenburg Center for Regenerative Therapies, Charité-Universitätsmedizin Berlin, Berlin, Germany; 4 Department of Comparative Medicine, King Faisal Specialist Hospital and Research Centre, Riyadh, Kingdom of Saudi Arabia; 5 Prince Naif Health Research Center, King Saud University, Riyadh 11461, Kingdom of Saudi Arabia

## Abstract

Regenerative medicine is a novel approach for treating conditions in which enhanced bone regeneration is required. We identified transgelin (*TAGLN*), a transforming growth factor beta (TGF*β*)-inducible gene, as an upregulated gene during *in vitro* osteoblastic and adipocytic differentiation of human bone marrow-derived stromal (skeletal) stem cells (hMSC). siRNA-mediated gene silencing of *TAGLN* impaired lineage differentiation into osteoblasts and adipocytes but enhanced cell proliferation. Additional functional studies revealed that TAGLN deficiency impaired hMSC cell motility and *in vitro* transwell cell migration. On the other hand, TAGLN overexpression reduced hMSC cell proliferation, but enhanced cell migration, osteoblastic and adipocytic differentiation, and *in vivo* bone formation. In addition, deficiency or overexpression of TAGLN in hMSC was associated with significant changes in cellular and nuclear morphology and cytoplasmic organelle composition as demonstrated by high content imaging and transmission electron microscopy that revealed pronounced alterations in the distribution of the actin filament and changes in cytoskeletal organization. Molecular signature of TAGLN-deficient hMSC showed that several genes and genetic pathways associated with cell differentiation, including regulation of actin cytoskeleton and focal adhesion pathways, were downregulated. Our data demonstrate that TAGLN has a role in generating committed progenitor cells from undifferentiated hMSC by regulating cytoskeleton organization. Targeting TAGLN is a plausible approach to enrich for committed hMSC cells needed for regenerative medicine application.

Regenerative medicine through employing stem cell transplantation is a novel approach for treating conditions in which enhanced bone regeneration is required. A number of stem cell types have been envisaged as candidates for use in therapy. Human bone marrow-derived stromal (also known as skeletal or mesenchymal) stem cells (hMSCs) is one of the most promising candidates. Optimal use of hMSC in therapy requires detailed understanding of molecular mechanisms of lineage commitment and differentiation as well as identifying regulatory factors that can be targeted for controlling hMSC differentiation and functions.

Global hypothesis generating methods, for example, DNA microarrays, proteomic analysis, and miRNA microarrays have been employed by our group in order to identify factors relevant to hMSC biology and functions and that exhibit significant changes during lineage-specific differentiation.^1, 2, 3, 4, 5^ This approach has led to the identification of several factors that control osteoblast or adipocyte differentiation of hMSC.^3^ Using transcriptomic profiling of differentiating hMSC, we identified transgelin (*TAGLN*) as a highly upregulated gene in hMSC during osteoblast and adipocyte differentiation.

TNGLN is a transforming growth factor beta (TGF*β*)-inducible gene^6^ that functions as an actin-crosslinking/gelling protein of the calponin family. It is localized to the cytoskeleton and is expressed by endothelial, smooth muscle cells, fibroblasts as well as several immune cells.^7^ It has several names: mouse p27, *WS3-10*, and *SM22*.^8^ It is transiently expressed in heart and skeletal muscle cells during early mouse embryogenesis.^9^ It is known as one of the earliest commitment markers of differentiated smooth muscle cells,^10, 11, 12^ and has been suggested to regulate their contractile functions. TAGLN-deficient mice were fertile and developed normally;^13^ however, vascular smooth muscle cells exhibited a pronounced alterations in the distribution of the actin filament and changes in cytoskeletal organization.^14^

The aim of this study was thus to examine the novel biological role for TAGLN in regulating hMSC lineage-specific differentiation and functions. We employed a number of cellular and molecular approaches including loss-of-function and gain-of-function studies. Our data suggest that TAGLN is important for differentiation progression of hMSC through regulation of distribution of actin filaments and cytoskeletal organization.

## Results

### Activation of TGF*β*1 signaling induces TAGLN expression and enhances hMSC differentiation

**Figure 1:**
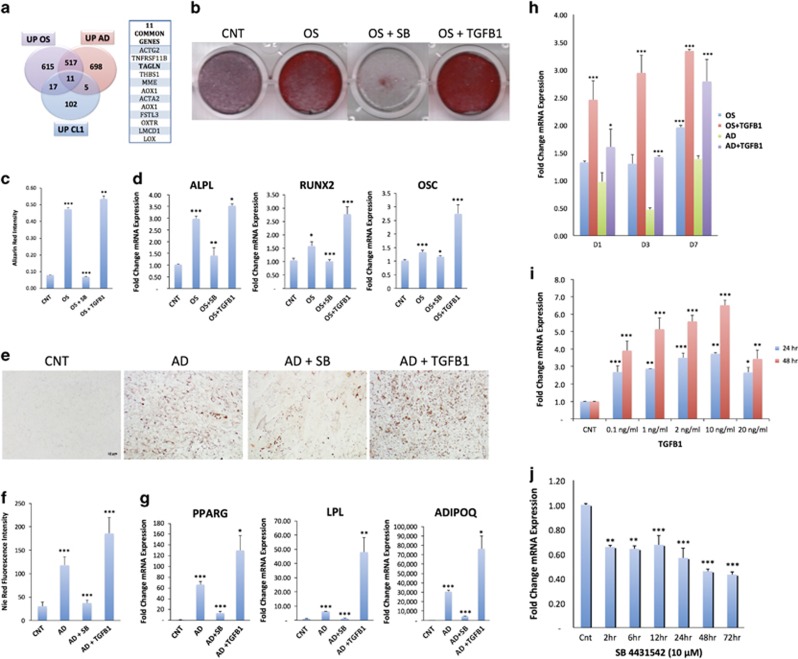
Activation of TGF*β*1 signaling induces TAGLN expression and enhances hMSC differentiation. (**a**) Venn diagram depicting the overlap between the differentially upregulated genes during osteogenic differentiation of hMSC, the upregulated genes during adipogenic differentiation of hMSC and the upregulated genes of CL1 *versus* CL2 cells. CL1 cells were differentiated into osteoblasts by osteogenic mixture for 7 days. (**b**) Mineralized matrix stained by Alizarin Red S (× 20, magnification). (**c**) Quantification of Alizarin Red S staining: control non-induced culture (NI), osteoblast-induced cultures (OS), with TGF*β*1 (10 ng/ml) or SB 431542 (10 *μ*m). (**d**) qRT-PCR performed for osteogenic markers, including *ALPL, RUNX2,* and *OSC*. Cells were differentiated into adipocytes by adipogenic induction mixture for 7 days. (**e**) Oil red-O staining of mature lipid-filled adipocytes ( × 20, magnification). (**f**) Quantification of Nile Red staining of mature lipid-filled adipocytes: non-induced controls (NI), adipocyte-induced (AD), with TGF*β*1 (10 ng/ml), or SB 431542 (10 *μ*m). (**g**) qRT-PCR for adipogenic markers: *LPL*, aP2 (adipocyte protein 2), *PPARG*, and *ADIPOQ*. (**h**) qRT-PCR of gene expression of *TAGLN* gene following osteogenic and adipogenic induction: D0 (non-induced), D1, D3, and D7 with and without TGF*β*1 induction. D=day. (**I**) Time- (24 and 48 h) and dose- (TGFB1: Control, 0.1, 1, 2, 10, and 20 ng/ml) response induction of *TAGLN* gene expression following treatment with TGFB. (**j**) Time-response suppression of *TAGLN* gene expression in response to SB 431542 (SB). Expression of target gene was normalized to GAPDH. Data are shown as mean±S.D. of three independent experiments, *P<0.05; **P<0.01, ***P<0.005

We have identified *TAGLN* as one out of 11 genes that were upregulated during osteogenic differentiation and adipogenic differentiation of hMSC as well as enriched in the hMSC clone 1 high osteogenic cell (CL1) cell line, which is an hMSC cell line that exhibits enhanced osteogenic and adipogenic differentiation (Figure 1a). We chose TAGLN as its role in regulating hMSC differentiation has not been investigated. Given the known role of TGF*β* signaling in regulating TAGLN expression, we subsequently assessed the effect of TGF*β* treatment on TAGLN expression and hMSC differentiation. Adding TGF*β*1 (10 ng/ml) to osteoblast induction medium enhanced osteoblast differentiation as evidenced by increased extracellular mineralized matrix formation (Figures 1b and c) and expression of osteoblast lineage gene markers: *RUNX2 (*runt related runt related transcription factor 2*), ALPL (*alkaline phosphatase, liver/bone/kidney*)* and osteocalcin (*OC;* Figure 1d). Similarly, TGF*β*1 enhanced adipocytic differentiation as evidenced by increased formation of mature lipid-filled adipocytes (Figures 1e and f) and expression of adipocyte lineage gene markers: *PPARG2 (*peroxisome proliferator activated receptor gamma*), LPL (*lipoprotein lipase*)*, and adiponectin (*ADIPOQ*; Figure 1g). Adding TGF*β*1 signaling inhibitor SB 431542 (SB; 10 *μ*m) to the induction media abolished TGF*β*1-stimulatory effect (Figures 1b–g). As seen in Figure 1h, the enhanced osteoblast and adipocyte differentiation by TGF*β*1 treatment was associated with increased expression of TAGLN. Dose–response experiments revealed induction of TAGLN by TGF*β*1 in a concentration range 0.1–20 ng/ml following 24 or 48 h treatment (Figure 1i). The specificity of TGF*β*1 induction of TAGLN was confirmed by adding SB 431542 (10 *μ*m) to the culture media for variable time (2 h–72 h) that resulted in inhibition of TAGLN expression already after 2 h treatment and with a maximal effect observed after 24 h and it continued for 72 h (Figure 1j). We confirmed the induction of TAGLN by TGF*β*1 and inhibition by SB using western blot analysis (Supplementary Figure S1A). To investigate whether the TGF*β*1-induced TAGLN expression is mediated by the SMAD2/3 pathway, we tested phosphorylation levels of SMAD2 and found that TGF*β*1 treatment increased phospho-SMAD2 levels (Supplementary Figure S1B).

### TAGLN-depleted cells exhibited impaired osteoblast and adipocyte differentiation

**Figure 2:**
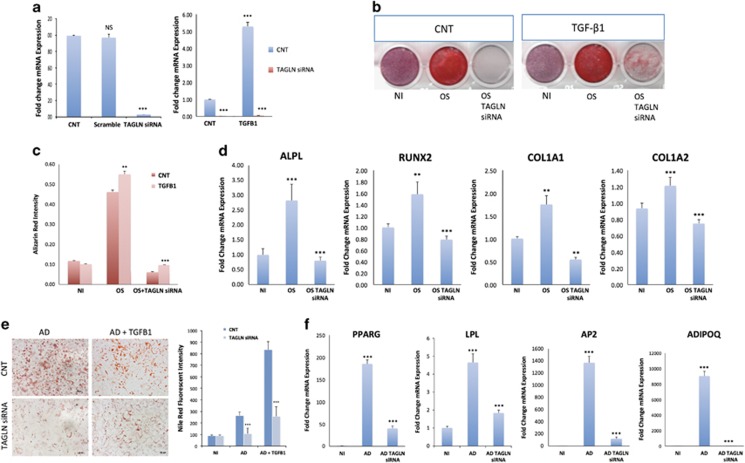
siRNA-mediated TAGLN suppression inhibits osteogenesis and adipogenesis. (**a**) Quantitative qRT-PCR for *TAGLN* gene expression 3 days post-TAGLN-siRNA, or scramble-siRNA transfection. Data are presented as fold induction. All further controls represent scramble-transfected cells. (**b**) Alizarin Red S staining for mineralized matrix formation. (**c**) Quantification of Alizarin Red staining: NI, OS, and TAGLN-siRNA cells cultured in osteoblast induction media in presence or absence of TGF*β*1. (**d**) qRT-PCR of gene expression of osteogenic markers (*ALPL, RUNX2, COL1A1*, and *COL1A2)*: NI, OS, and TAGLN-siRNA cells cultured in osteoblast-induction media. (**e**) Cells were induced using adipocyte induction mixture for 5 days and stained by Nile red for quantification of mature lipid-filled adipocytes: NI, AD, and adipocyte-induced cultures in presence of TGF*β*1 (AD+TGF*β*1) in control cells or cells transfected with TAGLN-siRNA. (**f**) qRT-PCR was performed for adipogenic markers' gene expression: *PPARG, LPL*, AP2 (adipocyte protein 2), and *ADIPOQ*: NI, AD, and adipocyte-induced cultures in presence of TGF*β*1 (AD+TGF*β*1). Expression of each target gene was normalized to GAPDH. Data are presented as mean ±S.D. from three independent biological samples from at least two independent experiments. ** *P*<0.01, ***P<0.005

As the enhanced osteoblastic and adipocytic differentiation by TGF*β*1 was associated with increased TAGLN expression, we employed a loss-of-function approach to assess the role of TAGLN in the differentiation processes. Small interfering RNA (siRNA)-targeting *TAGLN* downregulated *TAGLN* gene expression (Figure 2a) even in the presence of TGF*β*1 (10 ng/ml; Figure 2a). Western blot analysis confirmed suppression of TAGLN expression at the protein level (Supplementary Figure S1C).

As shown in Figure 2b, TAGLN-siRNA cells exhibited impaired osteoblast differentiation demonstrated by significant reduction in mineralized matrix formation in absence or presence of TGF*β*1 treatment (10 ng/ml; Figure 2b) that was confirmed by alizarin red quantification (Figure 2c), and decreased expression of osteoblastic gene markers: RUNX2, ALPL, COL1A1 (collagen type I alpha), COL1A2, periostin (POSTN), and DICKKOPF-related protein 2 (DKK2; Figure 2d and Supplementary Figure S1D). The following markers RUNX2, POSTN, and DKK2 were upregulated in the presence of TGF*β*1 and were significantly decreased in TAGLN-siRNA cells (Supplementary Figure S1D). Similarly, TAGLN-siRNA cells exhibited impaired adipocytic differentiation shown by decreased number of lipid-filled mature adipocytes quantified by Nile Red staining (Figure 2e) and decreased expression of adipocytic gene markers: PPARγ2, LPL, AP2, and ADIPOQ (Figure 2f). The effects of TAGLN deficiency on hMSC differentiation were further confirmed by establishing hMSC with a stable knockdown of *TAGLN* using shRNA (TAGLN-shRNA), where similar results were obtained (Supplementary Figure S2).

### TAGLN overexpression exhibited enhanced osteoblast and adipocyte differentiation of hMSC

**Figure 3:**
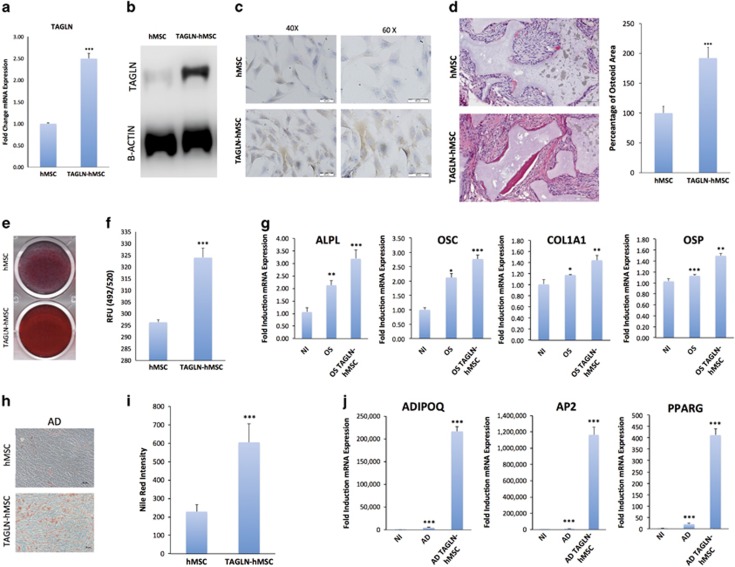
TAGLN overexpression induces osteogenesis and adipogenesis in hMSC. (**a**) All controls represent empty vector-transfected cells. qRT-PCR of *TAGLN* gene expression in control (empty vector) hMSC and TAGLN-overexpressing line (TAGLN-hMSC). (**b**) Western blotting for TAGLN in TAGLN-hMSC (upper panel) and B-Actin (lower panel). (**c**) TAGLN immunocytochemical staining in control hMSC and TAGLN-hMSC cells at magnification × 40 and × 60. Cells were differentiated into osteoblasts by osteogenic or adipogenic induction mixture for 14 days. (**d**) hMSC and TAGLN-hMSC cells were implanted with HA/TCP subcutaneously into NOD/SCID mice. The histology of *in vivo* formed bone was examined with H&E staining (original magnification × 200). High-resolution whole-slide digital scans of all histological sections were created with a ScanScope scanner and bone formed was quantified. (**e**) Mineralized bone matrix formation in control hMSC (empty vector) cells and TAGLN-hMSC stained for Alizarin Red. (**f**) Quantification of Alizarin Red staining in control hMSC and TAGLN-hMSC. (**g**) qRT-PCR for osteogenic markers: ALPL, OSC, COL1A1 (collagen type I), and OSP: NI, OS, and osteoblast-induced TAGLN-hMSC (OS TAGLN-hMSC). (**h**) Both control (empty vector) hMSC, and TAGLN-hMSC were induced to adipocyte differentiation using adipocyte induction medium for 7 days. Mature lipid-filled adipocytes were stained by oil red. (**i**) Quantification of Nile Red staining for mature adipocytes: control (empty vector) hMSC, TAGLN-hMSC. (**j**) qRT-PCR for adipogenic markers: ADIPOQ, LPL, AP2 (adipocyte protein 2), and PPARG in NI, AD, and adipo-induced TAGLN-hMSC line (AD TAGLN-hMSC). Data are shown as mean±S.D. from at least two independent experiments, *P<0.05; ** *P*<0.01, ***P<0.005

We established a TAGLN stably overexpressing hMSC-TERT (TAGLN-hMSC) by lentiviral transduction. The overexpression of TAGLN was confirmed by quantitative real-time polymerase chain reaction (qRT-PCR; Figure 3a), western blot analysis (Figure 3b), and immunocytochemical staining (Figure 3c). To examine the *in vivo* differentiation capacity, TAGLN-hMSC cells were mixed with hydroxyapatite–tricalcium phosphate (HA/TCP) and implanted subcutaneously into non-obese diabetic/ severe combined immunodeficiency (NOD/SCID) mice. Histological analysis of the implants revealed significant increased formation of ectopic bone in TAGLN-hMSC, as assessed by twofold increase in quantification of newly formed bone of TAGLN-hMSC comparing with the control (Figure 3d). Following differentiation induction, TAGLN-hMSC exhibited enhanced differentiation to osteoblastic cells evidenced by increased Alizarin Red S staining for formed mineralized matrix and expression of osteogenic gene markers (Figures 3e–g). In addition, adipocyte differentiation was enhanced as shown by increased number of Nile Red-positive mature adipocytes and significant upregulation of adipocytic gene expression (Figures 3h–j).

### TAGLN-overexpressing hFFs exhibited enhanced osteoblastic and adipocytic differentiation

**Figure 4:**
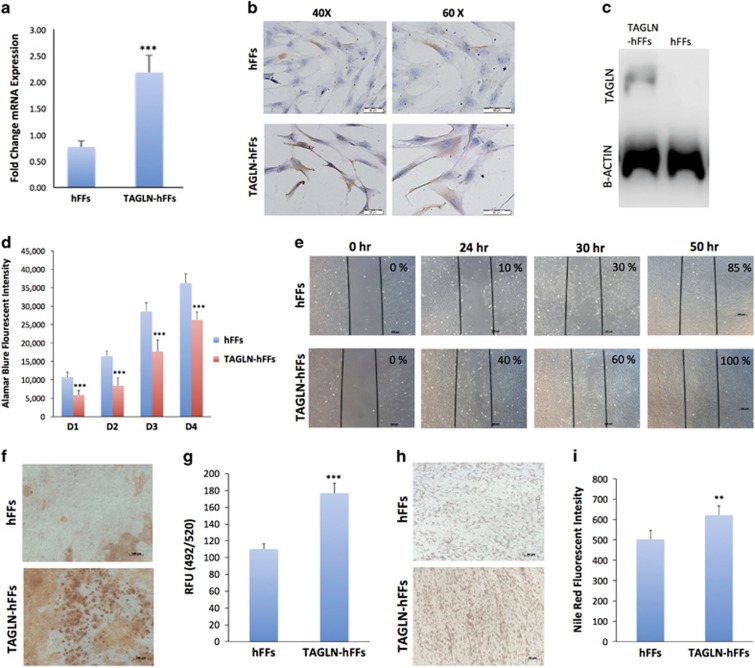
Effects of TAGLN overexpression on hFFs functions. (**a**) qRT-PCR of *TAGLN* gene expression in control (empty vector) TAGLN-hFFs. (**b**) Immunocytochemical staining for TAGLN protein in control hFFs, and TAGLN-hFFs at magnifications × 40 and × 60. (**c**) Western blot of TAGLN (upper panel) and B-Actin (lower panel) in control hFFs and TAGLN-hFFs. Cells were induced to differentiate into osteoblasts or adipocytes by either osteogenic or adipogenic mixture for 24 days. (**d**) Alamar blue staining for cell viability in control hFFs and TAGLN-hFFs on day (D): 1D, 2D, 3D, and 4D. (**e**) Scratch assay where the motility of hFFs and TAGLN-hFFs cells was observed at different time intervals: 0, 24, 30, and 50 h. (**f**) Alizarin Red S staining for mineralized matrix formation. (**g**) Quantification of mineralized matrix formation in control hFFs cells and TAGLN-hFFs using Osteo-Image Mineralization Assay. (**h**) Control hFFs and hFF-TAGLN were induced to adipocyte differentiation by incubation in adipocyte induction medium for 24 days and stained by oil red for lipid-filled mature adipocytes. (**i**) Quantification of Nile red staining of mature adipocytes formed in control hFF cells and TAGLN-hFFs. Data are shown as mean±S.D. of three independent experiments. **P<0.01, ***P<0.005

To confirm that TAGLN's osteogenic and adipogenic induction potential is not restricted to BM-derived hMSC, we sought to assess the role of TAGLN-hFFs).^4^ The TAGLN-hFF cells showed upregulation of *TAGLN* messenger RNA (mRNA) compared with control hFFs (Figure 4a), and increased protein expression as shown by immunocytochemical staining (Figure 4b) and western blot analysis (Figure 4c). TAGLN-hFFs exhibited decreased in proliferation rate, enhanced cell motility, and cell differentiation (Figures 4d–i).

### TAGLN inhibited hMS cell proliferation

**Figure 5:**
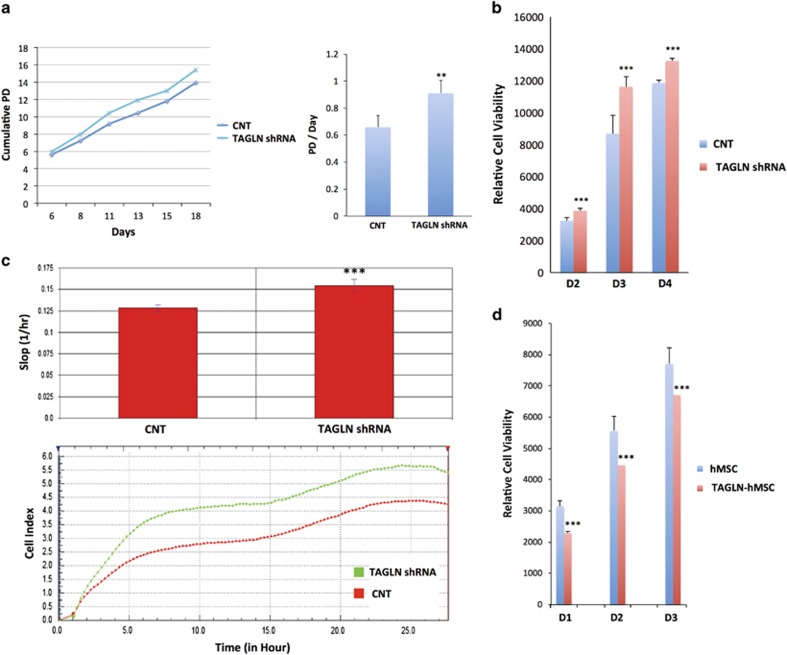
TAGLN inhibits cells proliferation. (**a**) Short-term growth curve showing cumulative PD over 18-day culture of control CL1 (CNT) and TAGLN-deficient cells (TAGLN-shRNA). (**b**) Alamar blue staining showing cell viability in control cells (CNT) and TAGLN-shRNA on days (D) 2, D3, and D4. (**c**) Proliferation assay using DIC device with the RTCA DP system. CNT and TAGLN-shRNA. Cell proliferation was measured at 15-min intervals for 27 h (lower panel). Upper panel shows cell proliferation slope during the 27 h observation period. (**d**) Alamar blue staining for control hMSC and TAGLN-overexpressing hMSC (TAGLN-hMSC) during 4 days of culture. Data are shown as mean±S.D. of at least two independent experiments. **P<0.01, ***P<0.005

To study the effects of TAGLN on hMSC on the overall changes in cell number, we employed one standard assay that measured cell viability, which is Alamar blue assay and a state-of-the-art real-time monitoring cell number using real-time cell analysis (RTCA, DP system). In addition, we applied growth curves based on direct cell counting and expressed as cumulative population doublings (PDs) over 18 days of culture, in which TAGLN*-shRNA* exhibited higher PD rate compared with control cells (Figure 5a). RTCA DP system and Alamar blue assays showed increased proliferation in TAGLN-*shRNA* (Figures 5b and c). On the other hand, TAGLN-hMSC exhibited decreased cell viability (Figure 5d).

### TAGLN enhanced hMSC migration

**Figure 6:**
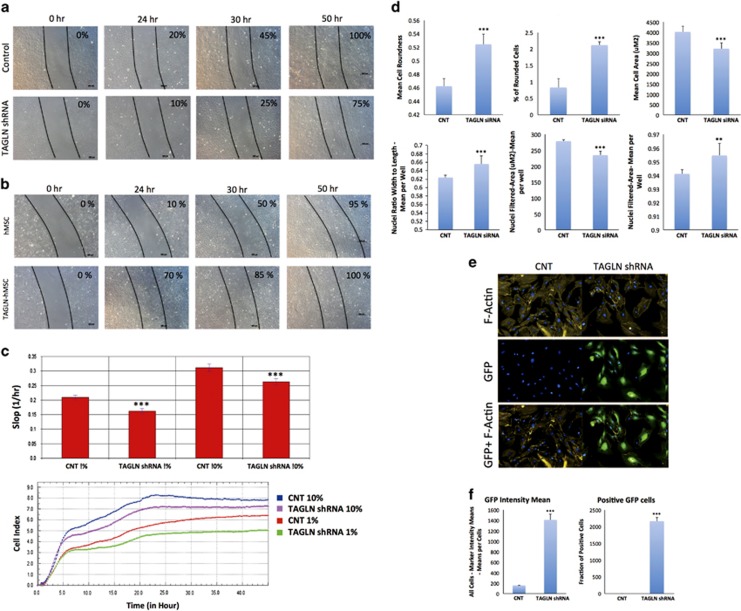
TAGLN induces changes in cell morphology and enhances transwell cell migration. Scratch assay where a scratch is made in 50–70% confluent cells and cell motility is observed at 0, 24, 30, and 50 h in (**a**) control CL1 (control) and TAGLN-deficient cells (TAGLN-shRNA), and (**b**) control hMSC and TAGLN-overexpressing hMSC (TAGLN-hMSC). (**c**) Transwell migration assay using DIC device with the RTCA DP system. FBS was used as chemoattractant. The curves show the number of cell migrated up to 40 h (lower panel). Control empty vector cells (CNT) and TAGLN-shRNA cultured in presence of 1 or 10% FBS. The cell migration was measured at 15 min intervals for 45 h. Upper panel shows the average cell migration slope during 24 h. (**d**) High content image analysis using Opretta of cell morphology in TAGLN-shRNA as compared with CNT. (**e**) Immunocytochemical staining for F-Actin protein in CNT and TAGLN-shRNA. (**f**) High content image analysis of GFP intensity and the number of GFP-positive cells in CNT and TAGLN-shRNA

TAGLN has previously been implicated in cell migration;^6, 15, 16, 17, 18, 19^ however, the role of TAGLN expression in hMSC migration has not been studied. Thus, we studied the effects of TAGLN deficiency and overexpression on hMSC cell migration in two independent assays. In scratch assays, TAGLN-hMSC migrated faster and tended to close the scratch defect compared with control cells or TAGLN-shRNA (Figures 6a and b). We have also conducted transwell migration assay using the RTCA DP system that revealed impaired migration of TAGLN*-*shRNA compared with control cells (Figure 6c).

### TAGLN-shRNA cells exhibited cellular and nuclear morphological changes

The observed effects of TAGLN on hMSC cell migration prompted us to study its effects on cell morphology and cytoskeleton. For this, we employed high content imaging using Opretta of Phalloidin-fluorescein isothiocyanate (FITC) staining for F-actin. As shown in Figure 6, TAGLN-shRNA cells exhibited increased cell roundness, percentage of rounded cells, index of nuclei mean roundness, and nuclei width to length ratio, but reduced cell area and nuclear area (Figure 6d). F-Actin staining revealed decreased staining intensity in TAGLN-shRNA cells (Figure 6e).

**Figure 7:**
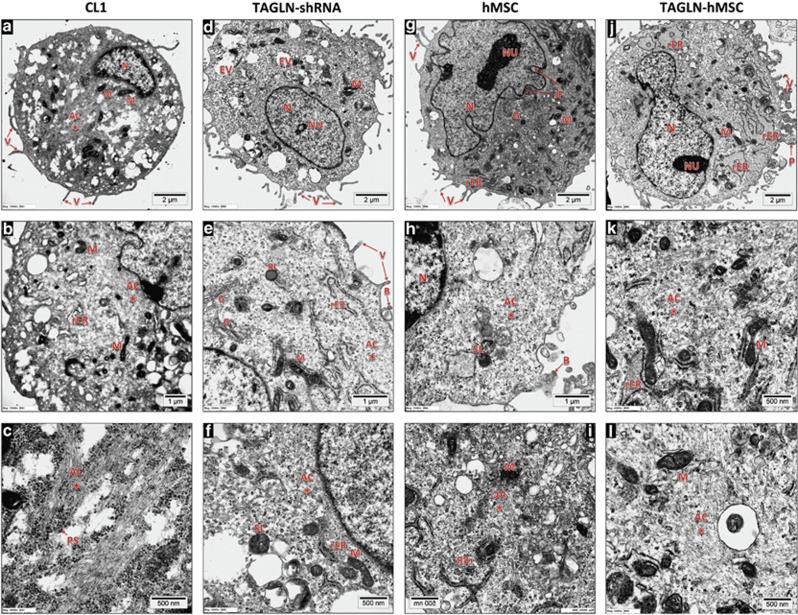
Transmission electron microscopy (TEM) analysis. Ultra-thin sections from resin embedded samples were ultrastructural analyzed using TEM. (**a**-**c**) Control empty vector (CL1) cells with magnification x12 000, x15 000 and x50 000 respectively. (**d**-**f**) TAGLN deficient cells (TAGLN-shRNA) with magnification x12 000, x25 000 and x40 000 respectively. (**g**-**i**) Control empty vector hMSC (hMSC) cells with magnification x12 000, x25 000 and x40 000 respectively. (**j**-**l**) hMSC overexpressing TAGLN (TAGLN-hMSC) cells with magnification x10 000, x25 000 and x40 000 respectively. AC, actin filaments; B, cell blebs; EV, endocytotic vacuole; G, Golgi bodies; IF, infolding of nuclear membranes; M, mitochondria; N, nucleus; Nu, nucleolus; P, cell processes; PL, primary lysosome; rER, rough endoplasmic reticulum; SL, secondary lysosome; V, microvilli

In addition, we employed transmission electron microscopic examination of TAGLN-shRNA cells and TAGLN-hMSC (Figure 7,Supplementary Figures S3–S6). In control cells, we observed prominent actin filament organized as bundles/aggregates and distributed in the whole cytoplasm and in perinuclear locations. In addition, the cells contained surface-branched microvilli. In contrast, TAGLN-shRNA contained relatively fewer actin filaments, whereas TAGLN-hMSC showed high content of actin filaments. Furthermore, rough endoplasmic reticulum (rER) was dilated in TAGLN-hMSC, suggesting increased protein synthesis activity. Interestingly, cellular processes were observed extending from TAGLN-hMSC that may enhance cellular motility. The ultrastructural characteristics of the examined cells are summarized in Supplementary Table C.

### Gene expression profiling of TAGLN-depleted cells

**Figure 8:**
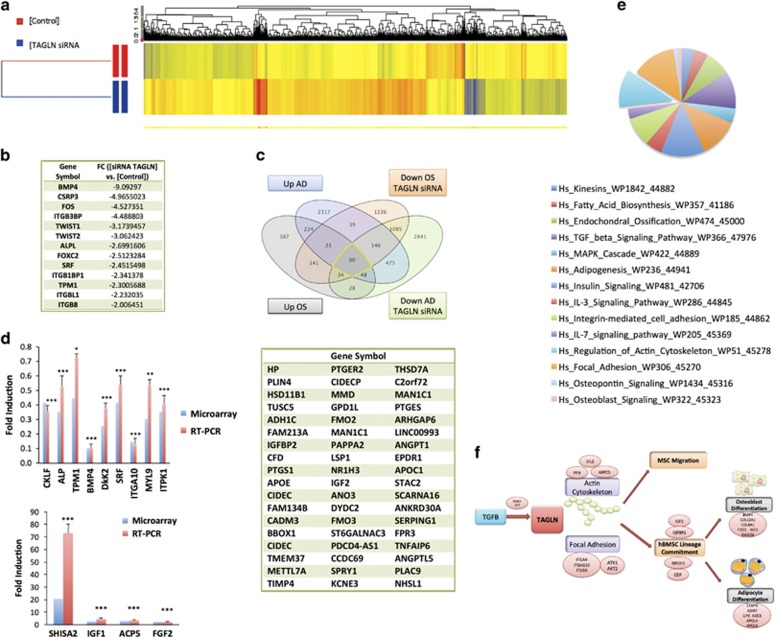
Gene expression profiling of TAGLN-depleted cells (TAGLN-siRNA). (**a**) Hierarchical clustering of control cells (control) and TAGLN-siRNA. Each row represents a biological sample and each column represents a transcript. Expression level of each gene in a single sample is depicted according to the color scale. (**b**) List of differentiation-associated genes that were downregulated in TAGLN-siRNA. (**c**) Venn diagram revealing overlap between the upregulated genes and the downregulated genes in TAGLN-siRNA following osteogenic and adipogenic differentiation, The list of the 60 common genes shown in the Venn diagram. (**d**) qRT-PCR validation of selected genes *P< 005; ***P< 0001. (**e**) Pie chart illustrating the distribution of the top pathways enriched in the downregulated genes in TAGLN-siRNA identified during osteoblast differentiation. (**f**) A working model illustrating the effect of TGF*β*-induced TAGLN expression on hMSC migration, and osteoblastic and adipocytic differentiation by modulating actin cytoskeletal organization and focal adhesion molecules and suggesting NRIH3, CDF, IGFBP2, and IGF2 genes as downstream targets

In order to determine the molecular mechanisms underlying TAGLN functions in hMSC biology, we performed global gene expression profiling in TAGLN*-*siRNA cells and control cells. Hierarchical clustering revealed that control and TAGLN*-*siRNA samples clustered separately (Figure 8a). We found that 6351 genes were upregulated and 3159 genes were downregulated in TAGLN*-*siRNA cells compared with control cells (fold change (FC)≥2.0, *P*<0.02, Supplementary Table F). Among the significantly downregulated genes were integrin family (*ITGA4, ITGA10,* and *ITGB1BP1*), *TPM1, Fos, SRF*, and *CSRP3* as well as mesodermal genes such as twist family (*TWIST1* and *TWIST2*), and MFH-1, *FoxC2* (Figure 8b). In addition, osteoblast-associated genes such as *ALPL* and *BMP4* were downregulated (Figure 8b). In addition, Venn diagram showed 60 common downregulated genes in TAGLN-deficient cells during osteoblast and adipocyte differentiation including *NR1H3, CDF, IGF2,* and *IGFBP2*, which could potentially have a role in commitment of hMSC to osteoblastic and adipocytic lineage (Figure 8c). As a confirmation of the microarray data, qRT-PCR showed good concordance between microarray and qRT-PCR for a selected gene panel and are known to be involved in cell differentiation and the TGF*β* pathway (Figure 8d).

### Pathway analysis of significantly regulated genes in TAGLN-siRNA cells

TAGLN*-siRNA* cells exhibited downregulation in several intracellular signaling pathways including pathways regulating actin cytoskeleton, focal adhesions (FAs), endochondral ossification, adipogenesis, TGF*β* signaling, and MAPK cascade (Figure 8e,Supplementary Figures S10–S13). Figure 8e shows a pie chart of the top significantly regulated signaling pathways in TAGLN-siRNA cells. Interestingly, 21 FA genes and 18 actin cytoskeleton-regulating genes were downregulated in TAGLN*-siRNA* cells including *AKT1, AKT2, PFN1, ARPC5, VIL2*, and integrins (Supplementary Table D).

### Gene expression profiling in TAGLN-siRNA during osteoblast differentiation

Transcriptomic profiling was conducted on CL1 control and *TAGLN*-depleted cells on day 3 of osteogenic differentiation. As shown in Supplementary Figure S7A, hierarchical clustering revealed clear separation of the control and TAGLN-siRNA samples. A list of the 17 osteogenic genes that were downregulated in TAGLN-siRNA cells is shown in Supplementary Figure S7B and includes *BMP4, COL12A1, CL8A1, FZD1, IGF2,* and *SMAD6*. Comparing the list of the downregulated genes in TAGLN-siRNA cells and the upregulated genes in the CL1 control cells, in the presence of osteogenic induction medium, identified 256 overlapped genes that are predicted to be involved in osteogenic differentiation (Supplementary Figure S7C). Furthermore, using significance analysis, 6004 up- and 2752 downregulated genes were identified in TAGLN-depleted cells during osteogenesis (2.0 FC, *P*<0.02, Supplementary Table F). Pathway analysis on the downregulated genes using GeneSpring GX revealed significant changes in several pathways related to osteogenesis including osteoblast signaling, osteopontin signaling, TGF*β*1 signaling, adipogenesis, endochondral ossification, and regulation of microtubule cytoskeleton (Supplementary Figure S7D).

### Gene expression profiling in TAGLN-depleted cells during adipocytic differentiation

Global gene expression profiling was conducted on parental and TAGLN-siRNA cells on day 3 of adipogenic differentiation. As shown in Supplementary Figure S8A, hierarchical clustering revealed clear separation of the CL1 control cells and TAGLN-siRNA cells. Using significance analysis, 2990 up- and 4717 downregulated genes were identified in TAGLN-depleted cells during adipogenesis (2.0 FC, *P*<0.02, Supplementary Table F). In addition, the adipogenic inhibition in TAGLN-siRNA cells was associated with downregulation of adipogenic markers including *FABP4, LPL, ADIRF, LIPE, APOL4, APOL6, AOC3*, and *CEBPA* (Supplementary Figure S8B). Comparing the list of the downregulated genes in TAGLN-siRNA cells and the upregulated genes in control cells, in the presence of adipogenic induction medium, revealed 729 common genes, which are predicted to be involved in adipogenic differentiation (Supplementary Figure S8C). Pathway analysis revealed significant enrichment in several pathways related to the TGF*β*1 signaling pathway, adipogenesis, fatty-acid biosynthesis, integrin-mediated cell adhesion, and cell differentiation (Supplementary Figure S8D).

## Discussion

Identifying regulatory factors that have a role in hMSC proliferation and differentiation is prerequisite for their optimal use in regenerative medicine protocols. In the present study, we unraveled novel roles for TAGLN in hMSC proliferation, migration, and differentiation. TAGLN was observed to be upregulated during hMSC lineage differentiation and in response to TGF*β* activation. TAGLN-mediated TGF*β*-dependent enhancement of lineage differentiation into osteoblasts and adipocytes inhibited hMSC cell proliferation and enhanced their migratory capacity. These effects are possibly mediated through changes in actin distribution and cytoskeleton dynamics.

We observed that TGF*β*1 upregulated TAGLN expression in hMSC, and these effects were specific, as they were abolished by TGF*β*-signaling inhibitor SB 431542. TAGLN expression was associated with increased SMAD phosphorylation. Previous studies have reported that TGF*β*1 upregulated TAGLN expression in a number of cell models, for example, smooth muscle cells, epithelial cells, neural crest stem cells Monc-1, and embryonic fibroblast cells (BALB 3T3).^6, 20, 21^ In addition, TGF*β*1-induced TAGLN expression is mediated by the SMAD2/3 signaling pathway.^6, 22^ Thus, TGF*β*-mediated TAGLN expression represents a general phenomenon.

Interestingly, adding TGF*β*1 to hMSC induction media induced both osteogenic and adipogenic differentiation, and these effects were dependent on TAGLN as they were abolished in TAGLN-deficient hMSC. TGF*β*1 exerts complex effects on hMSCs. It has been demonstrated that it regulates proliferation and differentiation of osteoprogenitor cells.^23, 24, 25, 26^ We observed that TAGLN exerted opposing effects on hMSC proliferation (inhibition) and differentiation (enhancement). Similar phenomenon has been observed in rat calvarial cultures where osteoblast differentiation coincided with decreased cell proliferation.^27^ It is plausible that TAGLN-mediated effects are based on increasing the committed precursor pool, thus allowing more cells to differentiate into osteoblasts and adipocytes. Taken together, we proposed that the molecular changes mediated via TGF*β*-induced TAGLN defines the biological process of hMSCs, and their osteogenic and adipogenic differentiation and proliferation potential.

In our studies, TAGLN enhanced hMSC migration. Similar to our findings, TAGLN silencing inhibited TGF*β*1-induced cell migration in lung fibrosis model.^6^ Interestingly, it has been hypothesized that TGF*β*1 released from the bone matrix during bone resorption phase of bone remodeling mediates coupling of bone resorption to bone formation by inducing migration of hMSC to the bone-formation sites.^28, 29^ It is plausible that the enhanced hMSC motility and migration is achieved through upregulation of TAGLN and may thus have a role in recruiting progenitor cells to the bone-formation sites.

TAGLN overexpression or deficiency resulted in significant changes in cell morphology, cytoskeleton, and functional capacity for migration. It has been reported that TAGLN is localized to cellular cytoskeleton and filamentous actin, which are the principal components of the cytoskeleton,^13, 30, 31, 32^ and thus can actively contribute to regulation of cell shape and cell polarity. The observed changes in cell morphology and cytoskeleton may have a role in hMSC differentiation. It has been reported that significant changes took place in the cytoskeleton during lineage commitment and differentiation of hMSC.^33, 34^ During hMSCs' lineage commitment, the cells undergo significant modifications in morphology and actin cytoskeletal organization, and this participates in cell-fate determination.^33^ In addition, during osteoblast differentiation, hMSCs morphologically change from fibroblast-like cells to cuboidal morphology, which is associated with changes in the cytoskeleton.^35^ Similarly, during differentiation of hMSCs to adipocytes, significant changes in cell morphology takes place.^36, 37^ Our data are in concordance with previous work showing that TGF*β*1 treatment suppresses MSC proliferation and participates in cytoskeleton changes and thus hMSC differentiation.^38^ Finally, osteoblast differentiation in response to mechanical stimulation is associated with alteration in actin cytoskeletal organization and is regulated by signals transmitted through membrane-associated adhesion molecules.^39^ The molecular mechanisms linking changes of the cytoskeleton and hMSC differentiation are under investigation. One of the extensively studied mechanisms involves Rho GTPase/Rho-associated Kinase, adhesion kinases, and extracellular matrix components that regulate Actin Depolymerization Factor (ADF) expression, for example, Destrin, Coflin1, and Coflin2.^40, 41, 42^ A recent study from our group has demonstrated that, during osteoblast differentiation of hMSCs, alteration in ADF expression leads to changes in the ratio of G-actin (monomer) and F-actin (polymerized), and inhibit actin depolymerization.^33^

Treating bone marrow-derived hMSCs with TGF*β*1 has previously reported to regulate differentiation potential of MSCs.^43^ On the basis of our study, we propose that the mechanism by which the TGF*β*1/TAGLN axis regulates hMSC differentiation into osteoblasts and adipocytes is through changes in the cell cytoskeleton. Given that TAGLN is an actin-crosslinking or actin-gelling protein,^9, 44^ our studies demonstrate that the structure of actin cytoskeleton determines the transition from undifferentiated to committed differentiating hMSC. It is also plausible that osteoblast/adipocyte-differentiated phenotype requires a special actin cytoskeleton structure, which is distinct from the cytoskeletal structure of proliferating undifferentiated cells.

Interestingly, our TEM studies have revealed that rER was cystically dilated in TAGLN-hMSC, suggesting an increased protein synthesis activity. It has been shown by TEM that the ultrastructure of human bone marrow hMSCs is rich in rER with dilated cisternae^45^ and that there is a reduction in the abundance of rER following osteoblast differentiation.^46^ Palomäki *et al.*^47^ have shown the presence of dilated rER in undifferentiated bone marrow hMSCs, whereas, after osteoblast differentiation, narrow rERs with more extracellular matrix were formed. Our data suggest that the presence of cystically dilated rER, which is an indication of hyperactivity of rER, is a prerequisite at the early stage of hMSC commitment to cell differentiation when enhanced protein synthesis is required, and this is accompanied with cytoskeletal changes, which regulate ER dynamics and activity. It is known that during mitosis the cisternal ER network changes its intracellular localization, because of cytoskeleton changes, to the peripheral membrane away from the mitotic spindle and chromosomes in order to not interfere with the segregation of chromosomes.^48, 49^ This might explain the increased cell proliferation in TAGLN-depleted cells, which constitute abundant of long-stalk cisternal rER.

It has been shown that cytoskeletal and FA corporate to influence the shape, mechanical properties and differentiation of MSCs.^50, 51, 52^ FAs (cell–matrix adhesions) are large macromolecular assemblies through which mechanical force and regulatory signals are transmitted between the extracellular matrix and an interacting cell.^53^ FA molecules such as ATK1, ATK2, and integrins (for example, ITGA10, ITGA4, and ITGB8) have an essential role in directing MSCs to osteogenic differentiation by changing the cytoskeleton that initiates extracellular matrix-induced osteogenic differentiation.^54, 55, 56^ Concordantly, *AKT1, AKT2, PFN1, ARPC5, VIL2*, and integrins were significantly downregulated upon TAGLN depletion, suggesting their implication in actin polymerization-mediated MSC differentiation.

In summary, we proposed a model (Figure 8f) where TGFB regulates TAGLN expression and this leads to enhanced MSC cell migration and osteoblast and adipocyte differentiation through modulating actin cytoskeletal organization and FA molecules. A number of possible molecules, for example, *NRIH3, CDF, IGFBP2, and IGF2*, are possibly implicated in this process. Our study thus provides a novel molecular insight into the role of the TGF*β* intracellular signaling pathway in bone and bone marrow adipose tissue formation. In order to control the lineage fate of hMSCs, TAGLN is postulated as a potential target for pharmacological intervention.

## Materials and Methods

### Cell culture

We have employed the hMSC-TERT cell line, which was created from primary normal human MSC by overexpressing human telomerase reverse transcriptase gene.^1, 4^ The hMSC-TERT cells have been extensively characterized, and they exhibit similar cellular and molecular phenotypes to primary MSC.^4^ For ease, we will refer to this cell line as hMSC for the remaining part of the manuscript. For the current experiments, we employed two subclones derived from hMSC called CL1 and hMSC clone 2 low osteogenic cell (CL2) that exhibits enhanced and reduced differentiation potential to osteoblasts and adipocytes, respectively. In addition, hFFs have been used to extend our functional studies of TAGLN. The cells were cultured in Dulbecco's Modified Eagle Medium (DMEM) supplemented with D-glucose 4500 mg/l, 4 mM l-Glutamine and 110 mg/l Sodium Pyruvate, 10% fetal bovine serum (FBS), 1 × penicillin–streptomycin (Pen–strep), and non-essential amino acids (all purchased from Gibco-Invitrogen, Waltham, MA, USA).

### siRNA-mediated transfection of hMSC

For transfection, CL1 cells in the logarithmic growth phase were reversed-transfected with Silencer Select Pre-designed TAGLN-siRNA (25 nM; Ambion, Carlsbad, CA, USA; ID: s13739 and Cat. No. 4392420; Ambion, The RNA Company, USA) using Lipofectamine RNAiMAX Reagent (Invitrogen, CA, USA) plus serum-free Opti-MEMI medium (Invitrogen) under the conditions described by the manufacturer. On day 3 of transfection, the cells were induced to osteogenic or adipogenic differentiation for an additional 1 week. Detailed information about siRNA reagents is described in Supplementary Table E.

### Establishing a stable TAGLN-deficient (TAGLN-shRNA) line

CL1 cells were seeded in 24-well plates for 1 day, followed by forward transfection by 50 *μ*l serum-free Opti-MEMI medium (Invitrogen) mixed with 0.5 *μ*l of Lipofectamine 2000 Reagent (Invitrogen). The cells were incubated for 5 min and 1 *μ*g of TAGLN-shRNA (GeneCopoeia, Inc., Rockville, MD, USA; Cat. No. HSH017877-2-CH1) was mixed with 50 *μ*l serum-free Opti-MEMI medium (Invitrogen) and incubated for 5 min. One hundred microliters of shRNA and lipofectamine mixture were added to 0.5 ml pen–strep-free DMEM per well. Transfection medium was changed next day with Pen–Strep-free DMEM and cultured for 3 days. Puromycin (1.5 mg/ml) was added when the cells reached confluence. A OmicsLink shRNA Expression vector was used (Cat. No.: HSH017877-1-CH1, OriGene Technologies (Rockville, MD, USA)) for knocking down TAGLN with Puromycin as selection marker and GFP as reporter gene. Detailed information about shRNA reagents is described in Supplementary Table E and Supplementary Figure S18.

### Establishing TAGLN-hMSC line

Lentiviral transduction was employed to create stable hMSC cell lines overexpressing TAGLN. Lentiviral transduction was conducted as previously described.^5^ Lentifect lentiviral particles encoding human TAGLN (LP-G0046-Lv213-200) or control lentiviral particles (LP-FLUC-LV105-0205) were purchased from GeneCopoeia Inc. Briefly, hundred thousand cells were seeded in DMEM in 24-well plate. Forty-eight hours later (~80 confluency), media were removed and then 20 *μ*l of lentiviral particles in 500 *μ*l of DMEM+5% heat-inactivated serum (Invitrogen) and 1% Pen–Strep supplemented with polybrene (8 *μ*g/ml; Sigma, St. Louis, MO, USA) were added to the cells. Seventy-two hours later, media were removed and transduced cells were expanded in T25 and selected using puromycin (1.5 mg/ml; Sigma).

### *In vitro* osteoblast differentiation

Cells were grown in standard DMEM growth medium in six-well plates at 0.3x10^6^ cells/ml. When 70–80% confluence, the cells were cultured in DMEM supplemented with osteoblast induction mixture containing 10% FBS, 1% Pen–strep, 50 *μ*g/ml l-ascorbic acid (Wako Chemicals, Neuss, Germany), 10 mm *β*-glycerophosphate (Sigma), and 10 nm calcitriol (1*α*,25-dihydroxy vitamin D3; Sigma), and 10 nm dexamethasone (Sigma). The media were replaced three times per week.

### *In vitro* adipocyte differentiation

Cells were grown in standard DMEM growth medium in six-well plates at 0.3 × 10^6^ cells/ml. At 90–100% confluence, cells were cultured in DMEM supplemented with adipogenic induction mixture containing 10% FBS, 10% horse serum (Sigma), 1% Pen–strep, 100 nm dexamethasone, 0.45 mm isobutyl methyl xanthine^57^ (Sigma), 3 *μ*g/ml insulin (Sigma), and 1 *μ*M Rosiglitazone^58^ (Novo Nordisk, Bagsvaerd, Denmark). The media were replaced three times per week.

### Cytochemical staining

#### Alizarin Red S staining for mineralized matrix

The cell layer was washed with PBS, and then fixed with 4% paraformaldehyde for 15 min at room temperature. After removing the fixative, the cell layer was rinsed in distilled water and stained with the 2% Alizarin Red S Staining Kit (ScienceCell, Research Laboratories, Cat. No. 0223) for 20–30 min at room temperature. Excess dye was washed off with water. For quantifying the Alizarin Red S staining, the Alizarin Red S dye was eluted in 800 *μ*l of acetic acid incubated in each well for 30 min at room temperature as described^59^ and measured in spectrophotometer (BioTek, Epoch) at 405 nm.

#### Osteo-image mineralization assay

The *in vitro* formed mineralized matrix was quantified using the Osteo-Image Mineralization Assay Kit (Lonza, Walkersville, MD, USA, Cat. No. PA-1503). Culture media were removed and cells washed once with PBS, and fixed with 70% cold ethanol for 20 min. Appropriate amount as recommended by the manufacturer of diluted staining reagent was added, and plates were incubated in dark for 30 min at room temperature. Cells were washed, and staining quantitation was performed using fluorescent plate reader at 492/520 excitation emission wavelengths.

#### Oil red-O staining for lipid droplets

Mature adipocytes filled with cytoplasmic lipid droplets were visualized by staining with Oil Red-O. After washing with PBS, the cells were fixed in 4% formaldehyde for 10 min at room temperature, and then rinsed once with 3% isopropanol and stained for 1 h at room temperature with filtered Oil Red-O staining solution (prepared by dissolving 0.5 g Oil red-O powder in 60% isopropanol). To quantify the mature adipocytes formed, Oil Red-O stain was eluted by adding 100% isopropanol to each well and color intensity was measured with spectrophotometer at 510 nm (Biotek Spectrophotometer, Epoch).

#### Nile red fluorescence determination and quantification of mature adipocytes

Stock solution of Nile red (1 mg/ml) in DMSO was prepared and stored at −20 °C protected from light. Staining was performed on fixed cells with 4% paraformaldehyde (Sigma) for 15 min. Cultured undifferentiated and differentiated cells were washed once with PBS. The dye was added directly to the cells (5 *μ*g/ml in PBS), and the cells were incubated for 10 min at RT. Fluorescent signal was measured using SpectraMax/M5 fluorescence spectrophotometer plate reader (Molecular Devices Co., Sunnyvale, CA, USA) using the bottom well-scan mode where nine readings were taken per well using excitation 485 nm and emission 572 nm spectra.

### Immunocytochemical staining

The cells were fixed in 4% paraformaldehyde (Sigma) for 15 min, and were permeabilized with 0.1% Triton X-100 (Sigma) for 10 min. To block nonspecific binding, cells were treated with 3% bovine serum albumin (Sigma) for 30 min, and then incubated with TAGLN antibody (ThermoFisher Scientific, Rockford, IL, USA, CAT# PA5-29767) diluted in PBS (1:100) at 4 °C overnight. After washing the cells with PBS, they were treated with secondary antibody (Goat polyclonal to anti-mouse and anti-rabbit IgG, Abcam) and incubated for 1 h at room temperature.

### Cell proliferation assays

#### Cell number

Cell number was determined after 2 or 3 days of culture. The cells were cultured in T25 tissue culture flask dish at cell density 0.5 × 10^6^ cells (28 000 cells/cm^2^). At confluence, the cells were trypsinized and counted manually with hemocytometer. The proliferation curve is shown as cell number at each passage against number of days in culture.

#### Alamar blue cell viability assay

Cell viability was measured using Alamar blue assay according to the manufacturer's recommendations (AbD Serotec, Raleigh, NC, USA). In brief, to cultured cells in 96-well plates 10 *μ*l of Alamar blue substrate was added and the plates were incubated in the dark at 37 °C for 1 h. Reading was subsequently taken using fluorescent mode (Ex 530 nm/Em 590 nm) using BioTek Synergy II microplate reader (BioTek Inc., Winooski, VT, USA).

#### RTCA cell proliferation assay

The RTCA DP system was used to measure the cell proliferation rate. Cells were cultured for 24 h in 1% FBS-DMEM, followed by adding 100 *μ*l 10% FBS-DMEM to each well. E-plate 16 (ACEA Biosciences Inc., San Diego, CA, USA, ID: 691315, China) was placed onto PTCA DP analyzer inside the 37 ^o^C incubator. After 1 h, the background measurements were performed in RTCA SW. This is followed by adding 5000 cells in 100 *μ*l 10% FBS-media per well to each well, and CIM plates containing the cells were placed in the DP system inside the incubator followed by measurements with 15 min intervals.

### Scratch assay

This assay was conducted as previously described.^44^ Overall, 0.2 × 10^6^/cells/dish were plated on 30-mm cell culture dish. At confluence, the cell monolayer was scraped in a straight line to create a 'scratch' with a p10 pipet head. The debris was removed and markings to be used as reference points close to the scratch were created by tip marker, and the plates were placed under the phase-contrast microscope. Images of the scratch were acquired, and then the plates were placed in the tissue culture incubator. After incubation, the plates were examined with a phase-contrast microscope, and the reference points were matched. The previously acquired photographed regions were aligned and additional images were acquired. To determine the rate of cell migration, lines were drawn along the edges of the scratch and the percentage of migrated cells at each time point was estimated.

### RTCA cell migration assay

RTCA migration assay measures the effect of perturbations in electrical impedance in a label-free real-time setting. Migration assay was applied using DIC device with the RTCA DP system using FBS as a positive chemoattractant control. After 24 h incubation in 1% FBS-DMEM, the bottom chamber wells of the RTCA Cim-16 plate (ACEA Biosciences Inc., ID: 368179) were filled with 160 *μ*l media (for each sample two wells were used, one filled with 1% FBS-media as a control and the other filled with 10% FBS-media as chemoattractant). The upper chamber was placed over the bottom one and filled with 25–50 *μ*l 1% FBS-media to cover the membrane surface. CIM plate was placed on PTCA DP analyzer inside the 37 ^o^C incubator for 1 h incubation to allow the CIM membrane surface to reach the equilibrium with the media. After 1 h the background measurement was performed in RTCA SW. Cells (40 000) in 100 *μ*l 1% FBS-media were added to each well of the top chamber. The CIM plates containing the cells were placed in the DP system inside the incubator, followed by the measurement with 15 min intervals.

### Western blotting

Whole-cell lysates were prepared as previously described. Soluble proteins were analyzed by immunoblotting with TAGLN antibody (ThermoFisher Scientific, CAT# PA5-29767, diluted 1 : 50 00), total SMAD, P-SMAD2 (Cell Signaling, Boston, MA, USA, Cat. No. 9523, diluted 1:500), and anti-*β*-ACTIN (Sigma, A3854, diluted 1:10 000). Reactivity was detected with horseradish peroxidase-conjugated secondary antibodies (Santa Cruz Biotechnology, Dallas, TX, USA) and Clarity western ECL substrate (Bio-Rad, Hercules, CA, USA) for chemiluminescence using C-Digit Blot Scanner (LI-COR).

### High content imaging using Opretta

Cells were plated in 96-cell carrier microtiter plates (Perkin Elmer) at 1500 cells/well in standard culture medium supplemented with 10% FCS at 37 °C. After 24 h, the cells were fixed in 4% paraformaldehyde for 10 min, washed with PBS and stained for F-actin with Phalloidin-FITC (Sigma) and for nuclear staining with DAPI. Fluorescent images were analyzed using the Operetta high content imaging system (Perkin Elmer, Waltham, MA, USA) at a × 20 magnification. Different parameters of cytoskeletal changes were measured by using Harmony High Content Imaging and Analysis Software (Perkin Elmer).

### Transmission electron microscopy

After trypsinization, the cells were washed with PBS and the cell pellets were resuspended in 2.5% glutaraldehyde fixative (Electron Microscopy Sciences, Hatfield, PA, USA, Cat. No. 16500) in 0.1 m phosphate buffer (pH 7.2) and kept at 4 °C for 4 h. The cells were washed in 0.1 m phosphate buffer (pH 7.2) and transferred to 1% osmium tetroxide (OsO_4_) in 0.1 m phosphate buffer (pH 7.2). The cells were dehydrated in ascending grades of ethanol (10, 30, 50, 70, 90, and 100%) for 15 min each. The cells were then resuspended in acetone for 15 min and were aliquoted into BEEM embedding capsules and infiltrated with an acetone: resin mixture. Polymerization of the resin was accomplished in an oven at 70 °C for 12 h. Semi-thin sections (0.5 *μ*m thickness) were prepared and stained with 1% Toluidine Blue. Ultrathin sections (70 nm thickness) were prepared and mounted on copper grids. Ultrathin sections were first contrasted with uranyl acetate (saturated ethanol solution) for 30 min, rinsed, contrasted with Reynold's lead citrate for 5 min, and finally rinsed with distilled water. The contrasted ultrathin sections were examined and photographed under a TEM (Jeol 1010, Jeol, Tokyo, Japan).^60^

### *In vivo* subcutaneous implantation studies

All animal experimental procedures were approved by the animal care and use committees of the University of South Denmark. Cells were harvested via trypsinization, washed in PBS, and resuspended in PBS. Approximately 5 × 10^5^ cells were mixed with 40 mg of hydroxyapatite–tricalcium phosphate ceramic powder per each implant (HA/TCP, Zimmer Scandinavia, Albertslund, Denmark) and implanted subcutaneously into the dorsal surface of 8-week-old female NOD/SCID mice (NOD/LtSz-Prkdcscid), as described in more detail previously.^61^ Three mice were used with two implants in each mouse. After 14 weeks, the implants were recovered, fixed in 4% paraformaldehyde, decalcified using formic acid solution (0.4 m formic acid and 0.5 m sodium formate), and embedded in paraffin.

### Histologic analyses

Tissue blocks were sectioned at 4 mm. Immunohistochemical staining was performed on implants using DAKO En Vision and PowerVision according to the manufacturer's instructions (DAKO, Glostrup, Denmark). Human-specific staining was performed for human Vimentin. Briefly, paraffin sections were incubated for 1 h at room temperature with primary antibodies diluted in ChemMate (DAKO). Sections were washed subsequently in Tris-buffered saline (TBS, 0.05 m, pH 7.4), incubated for 30 min with secondary anti-mouse Ig/HR-conjugated polymers (K4001, En Vision., DAKO), and visualized with 3.30 diaminobenzidine tetrahydrochloride (DAB, S3000, DAKO) according to the manufacturer's instruction. Controls were performed without addition of primary antibodies and processed under identical conditions.

### Image analysis methodology

High-resolution whole-slide digital scans of all histological sections were created with a ScanScope scanner (Aperio Technologies Inc., Buffalo Grove, IL, USA). Five images were captured for each section. The digital slide images were examined using the Aperio's ImageScope viewing software (Aperio Technologies Inc.). Random snapshots were taken from each section with × 10 objective magnification. Each snapshot measured 1 809 171.65 *μ*m^2^ (~1.8 mm^2^). The images were then subjected to image analysis using the ImageJ software (National Institute of Health). Each image was color-thresholded so as to select only the pink color of osteoid tissue (as stained with hematoxylin and eosin) and measured its area percentage relative to the total area of the image. The analysis output results were then exported to Excel sheets and subjected to statistical analysis.

### DNA microarray global gene expression profiling

Total RNA was extracted using PureLink RNA Mini Isolation Kit (Ambion by Life Technologies, USA, Cat. No.: 12183018A) as recommended by the manufacturer. One hundred and fifty nanograms of total RNA were labeled and then hybridized to the Agilent Human SurePrint G3 Human GE 8 × 60 k v16 microarray chip (Agilent Technologies, Santa Clara, CA, USA). All microarray experiments were conducted at the Microarray Core Facility (Stem Cell Unit, King Saud University College of Medicine). Normalization and data analyses were conducted using the GeneSpring GX software (Agilent Technologies). Pathway analysis was conducted using the Single Experiment Pathway analysis feature in GeneSpring 12.0 (Agilent Technologies) as described in (ref. 5). Twofold cutoff with *P*<0.02 was used.

### Quantitative real-time PCR (qRT-PCR)

Total RNA was extracted using the PureLink Kit (Ambion by Life Technologies, Cat. No.: 12183018A) as recommended by the manufacturer. Total RNA was quantified using Nanodrop spectrophotometer (Nanodrop 2000, ThermoScientific). Complementary DNA (cDNA) was synthesized from 1 *μ*g of the RNA using High Capacity cDNA Reverse Transcription Kit (Applied Biosystem (ABI), USA) using Labnet, Multigene themocycler according to the manufacturer's instructions. Relative levels of mRNA were determined from cDNA using real-time PCR (ABI-Real-Time PCR Detection System) with Power SYBR Green PCR Kit (ABI, UK), or with TaqMan Universal master Mix II, no UNG (ABI) according to the manufacturer's instructions. Following normalization to the reference gene glyceraldehyde 3-phosphate dehydrogenase (GAPDH), quantification of gene expression was carried out using a comparative Ct method where ΔCT is the difference between the CT values of the target and reference gene. Primers employed are listed in Supplementary Tables A and B.

### Statistical analysis

All of the results were presented as the mean and S.D. of at least three independent experiments. Student's *t*-test was used for testing differences between groups. *P*-values <0.05 was considered statistically significant.

## Abbreviations

TAGLN: Transgelin
TGF*β*: transforming growth factor beta
hMSC: human bone marrow-derived stromal (skeletal) stem cells
CL1: hMSC clone 1 high osteogenic cells
CL2: hMSC clone 2 low osteogenic cells
mRNA: messenger RNA
siRNA: small interfering RNA
qRT-PCR: quantitative real-time polymerase chain reaction
RUNX2: runt related runt related transcription factor 2
ALPL: alkaline phosphatase, liver/bone/kidney
MAPK: mitogen-activated protein kinase
GAPDH: glyceraldehyde 3-phosphate dehydrogenase
ABI: Applied Biosystem
OC: osteocalcin
ADIPOQ: adiponectin
PPARG2: peroxisome proliferator activated receptor gamma
LPL: lipoprotein lipase
COL1A: collagen type I alpha
POSTN: periostin
DKK2: DICKKOPF-related protein 2
HA/TCP: hydroxyapatite–tricalcium phosphate
NOD/SCID: Non-obese diabetic/ severe combined immunodeficiency
hFF: human foreskin fibroblast
PD: population doubling
RTCA: Real-time cell analysis
FITC: Fluorescein isothiocyanate

## Supplementary Material

Supplementary Table f

Supplementary Figure 12

Supplementary Table a

Supplementary Table b

Supplementary Table c

Supplementary Table d

Supplementary Table e
