# Supplementary figures and images for: Transgelin is a TGFβ-inducible gene that regulates osteoblastic and adipogenic differentiation of human skeletal stem cells through actin cytoskeleston organization
Source: Cell Death Dis. 2016 Aug 4;7(8):e2321–. doi: 10.1038/cddis.2016.196 (PMC5108308; doi:10.1038/cddis.2016.196)

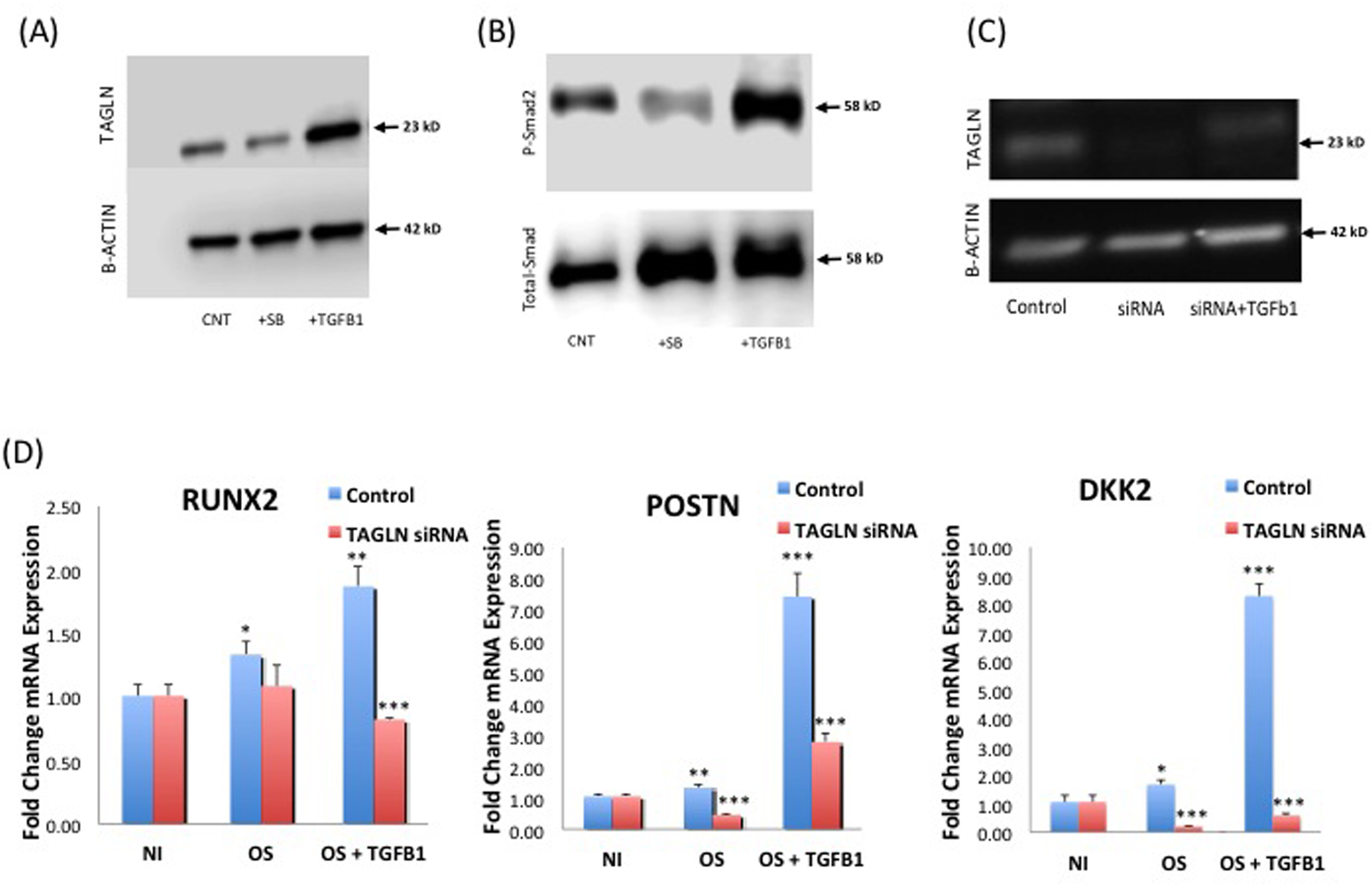

Supplement: Supplementary Table f [file cddis2016196x1.tif]

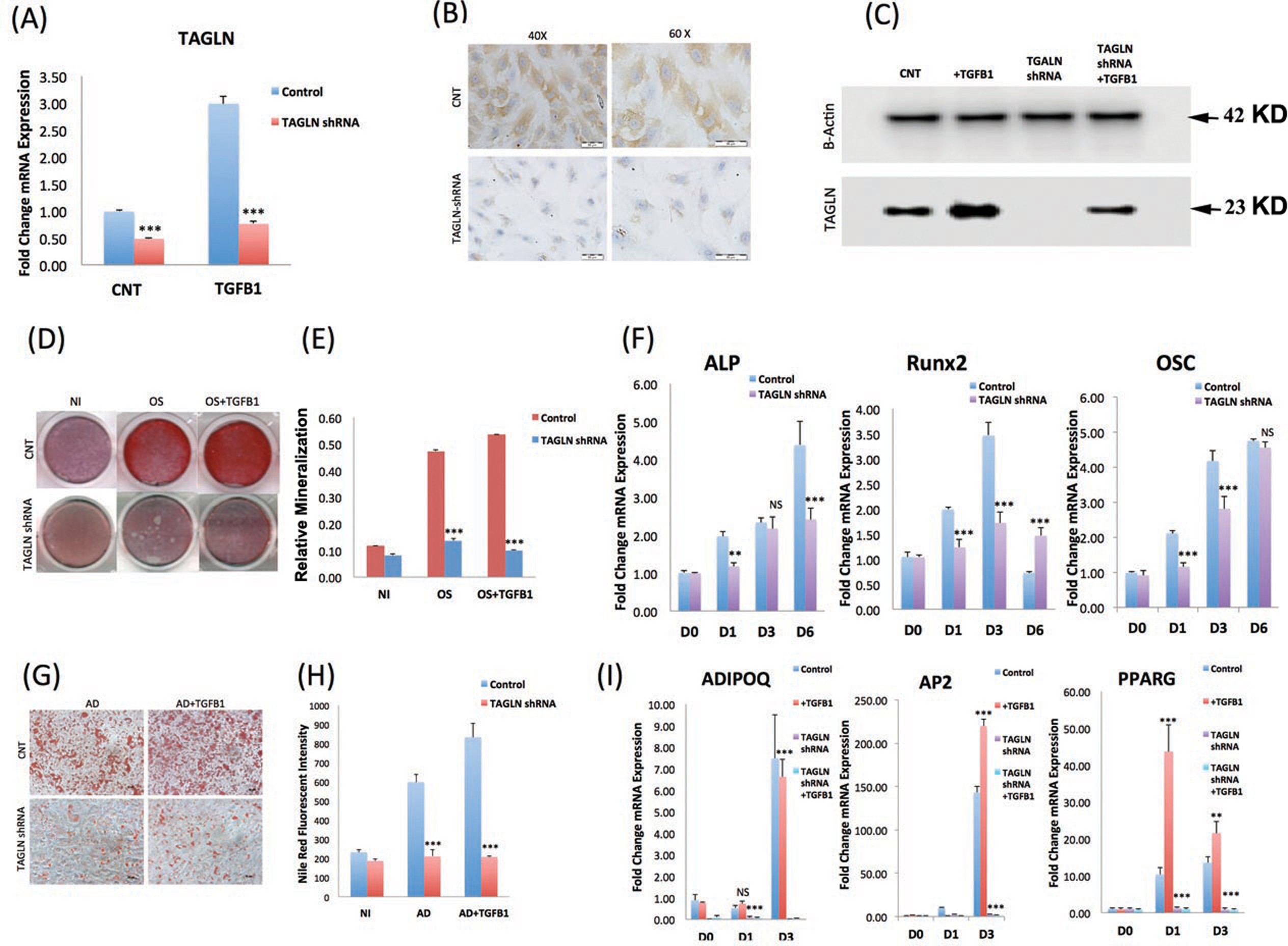

Supplement: Supplementary Figure 1 [file cddis2016196x2.tif]

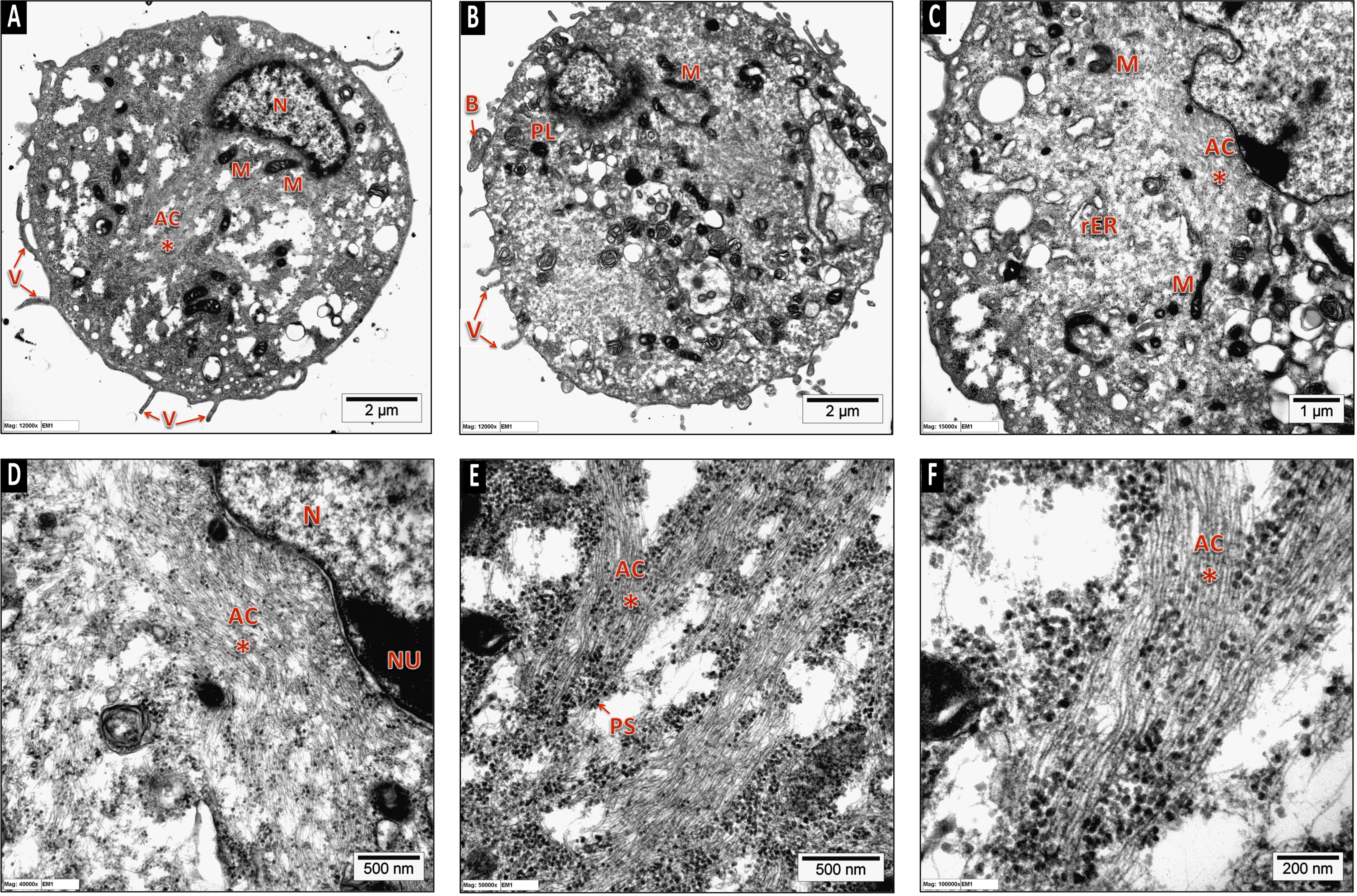

Supplement: Supplementary Figure 2 [file cddis2016196x3.tif]

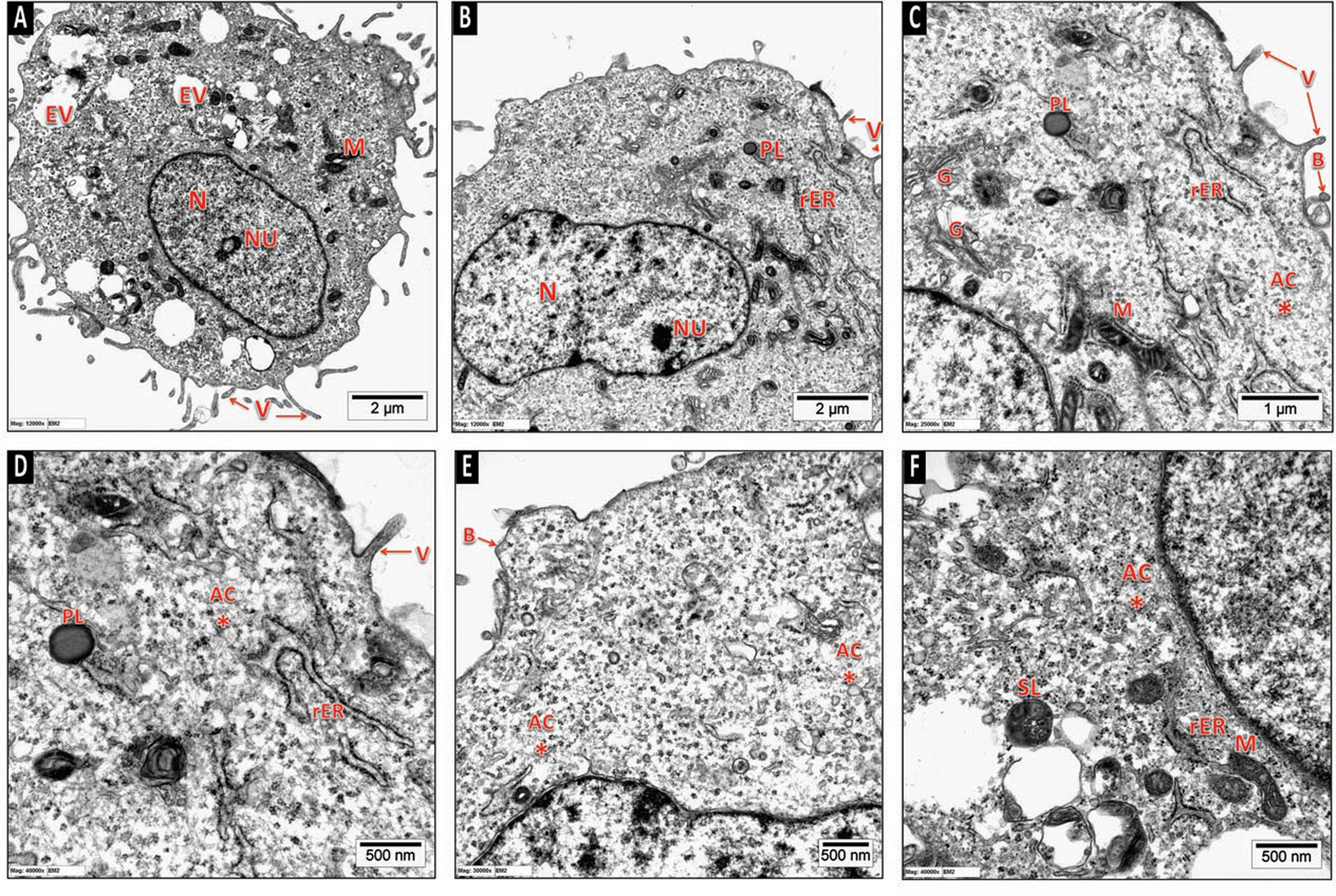

Supplement: Supplementary Figure 3 [file cddis2016196x4.tif]

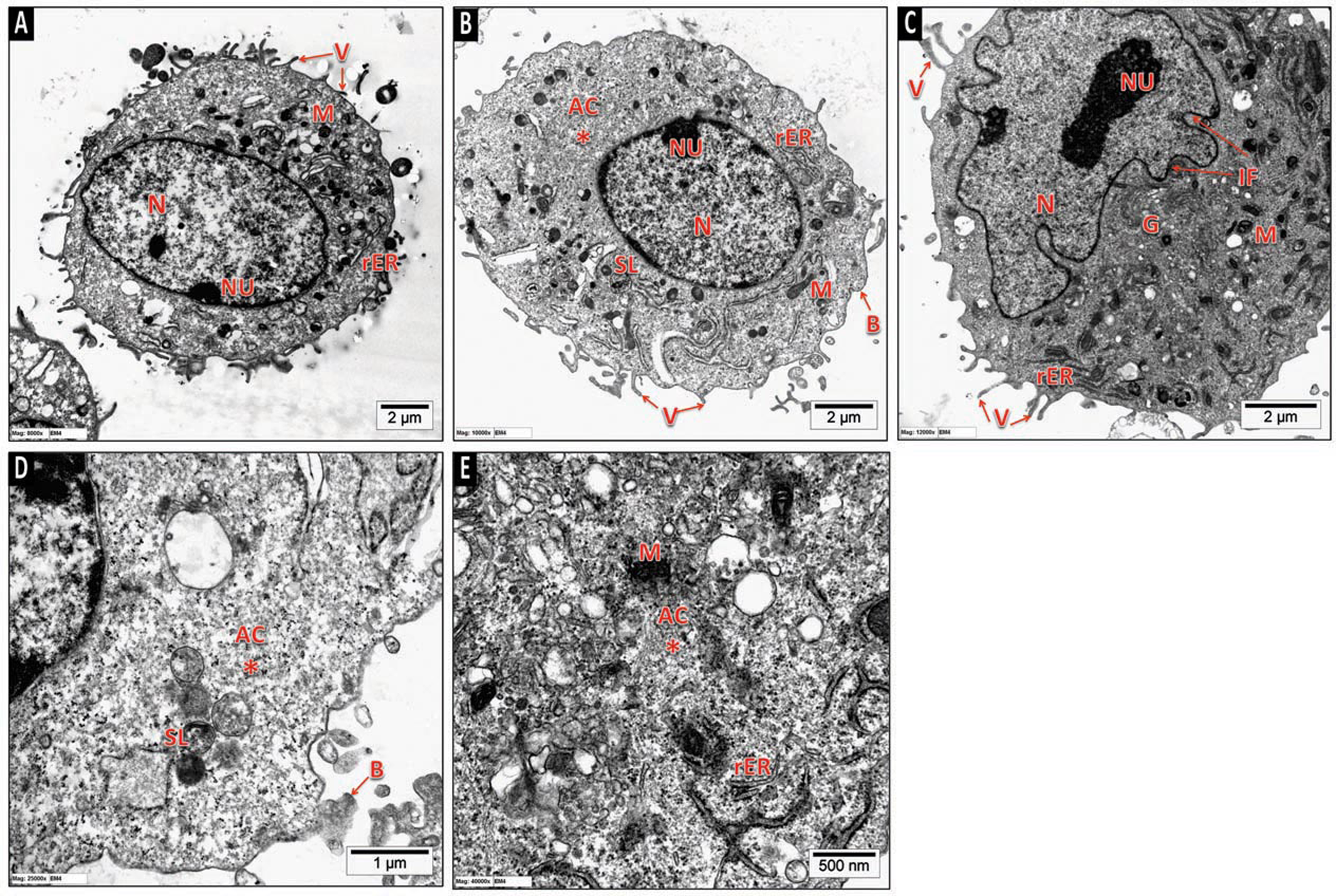

Supplement: Supplementary Figure 4 [file cddis2016196x5.tif]

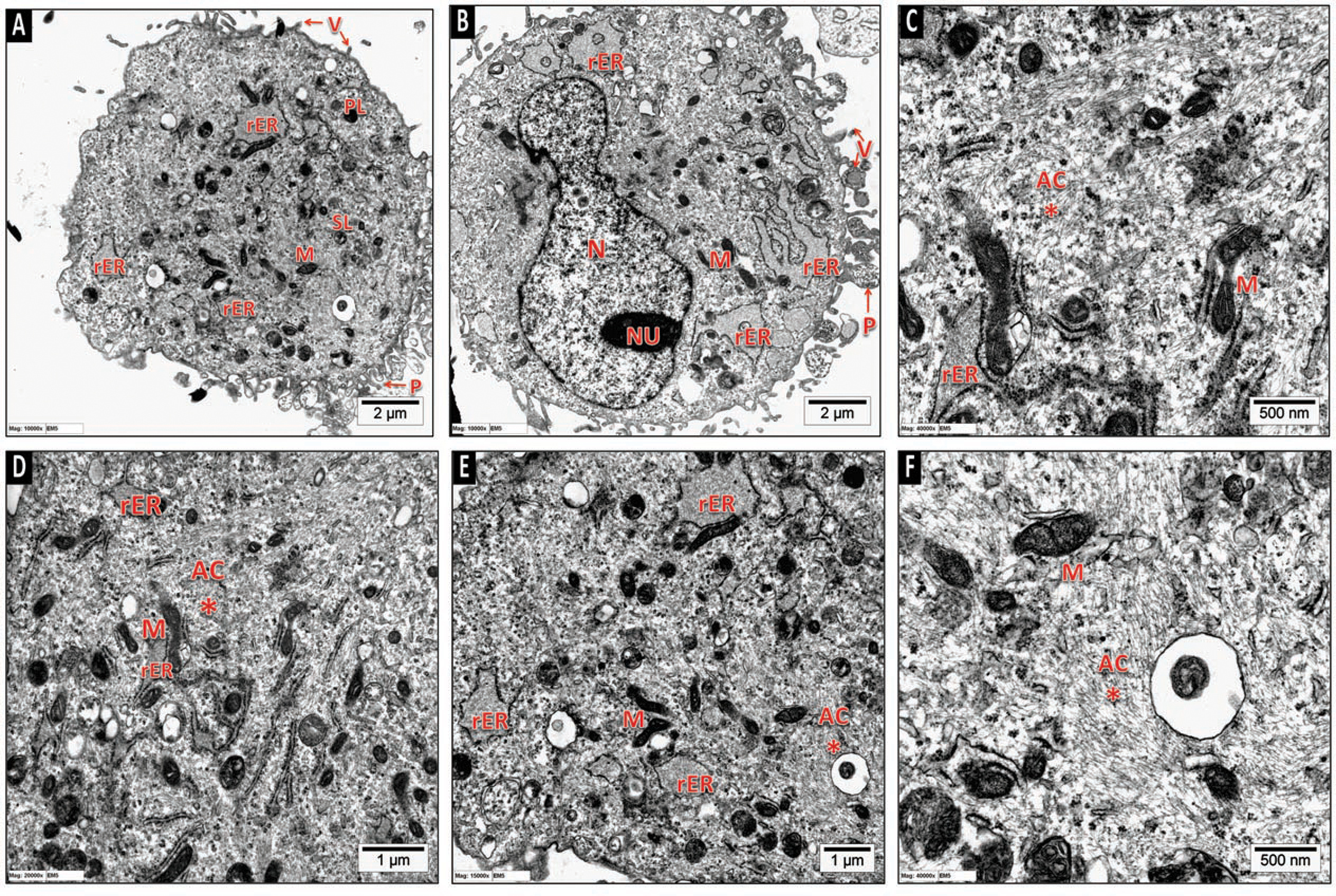

Supplement: Supplementary Figure 5 [file cddis2016196x6.tif]

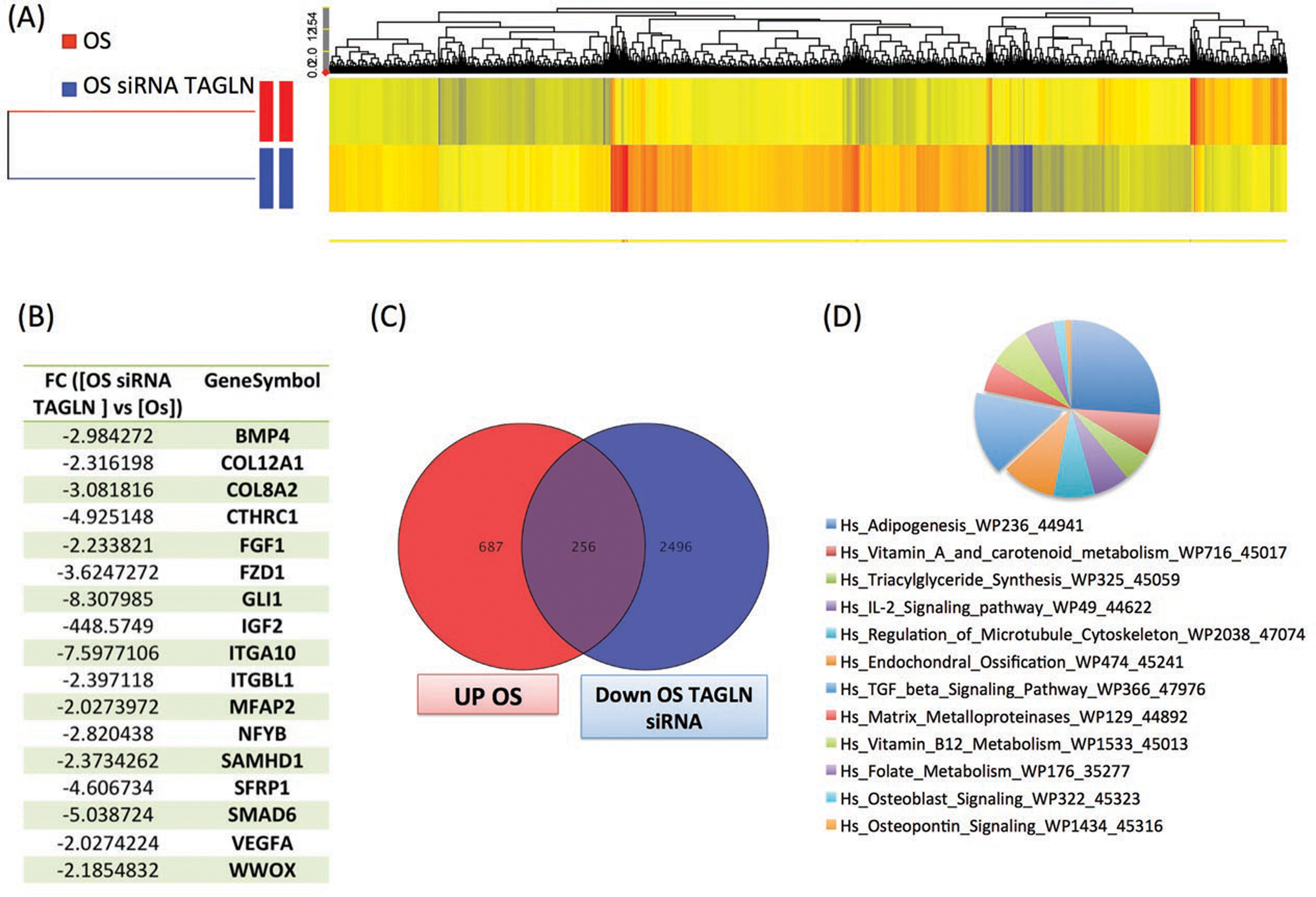

Supplement: Supplementary Figure 6 [file cddis2016196x7.tif]

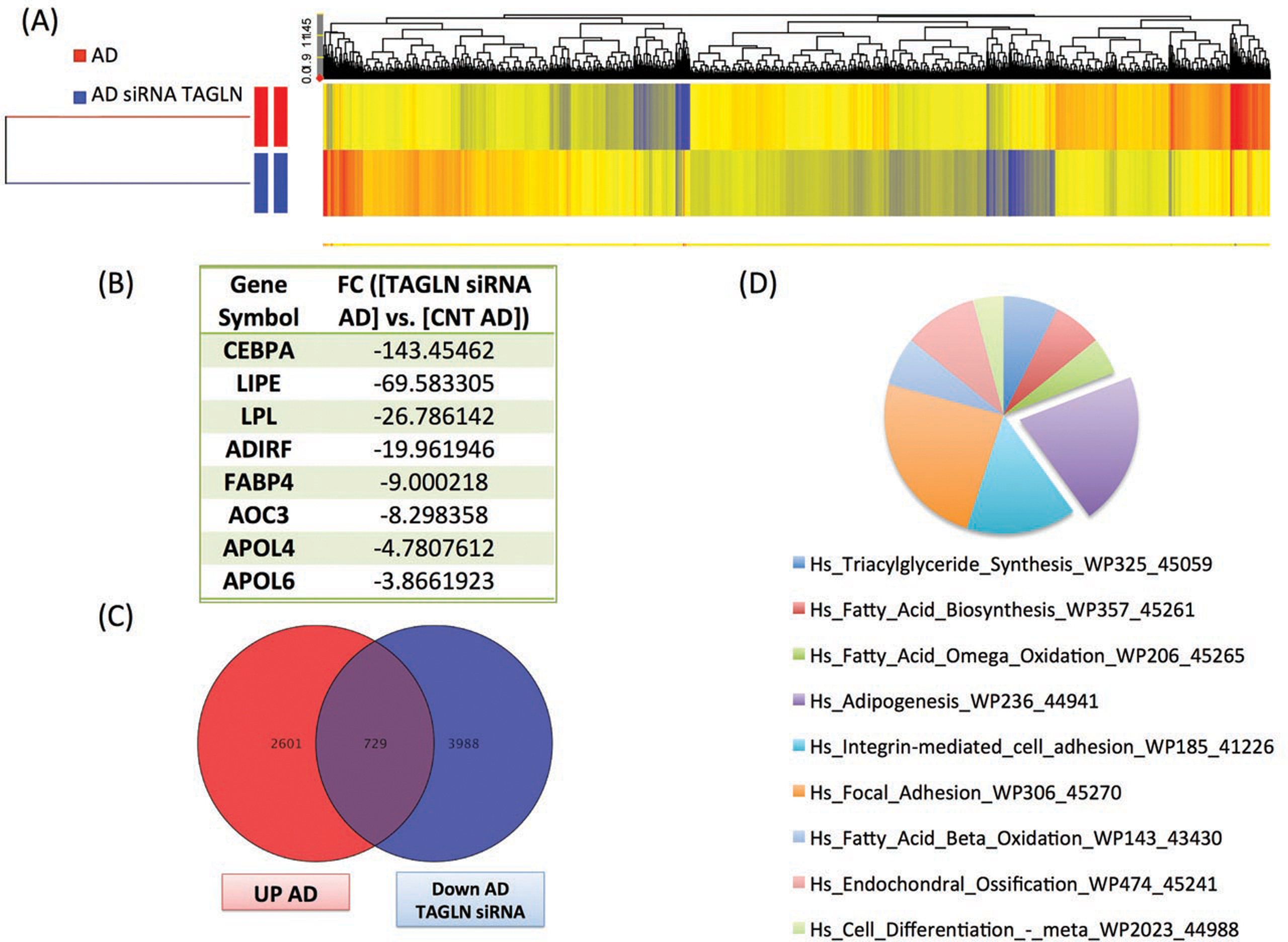

Supplement: Supplementary Figure 7 [file cddis2016196x8.tif]

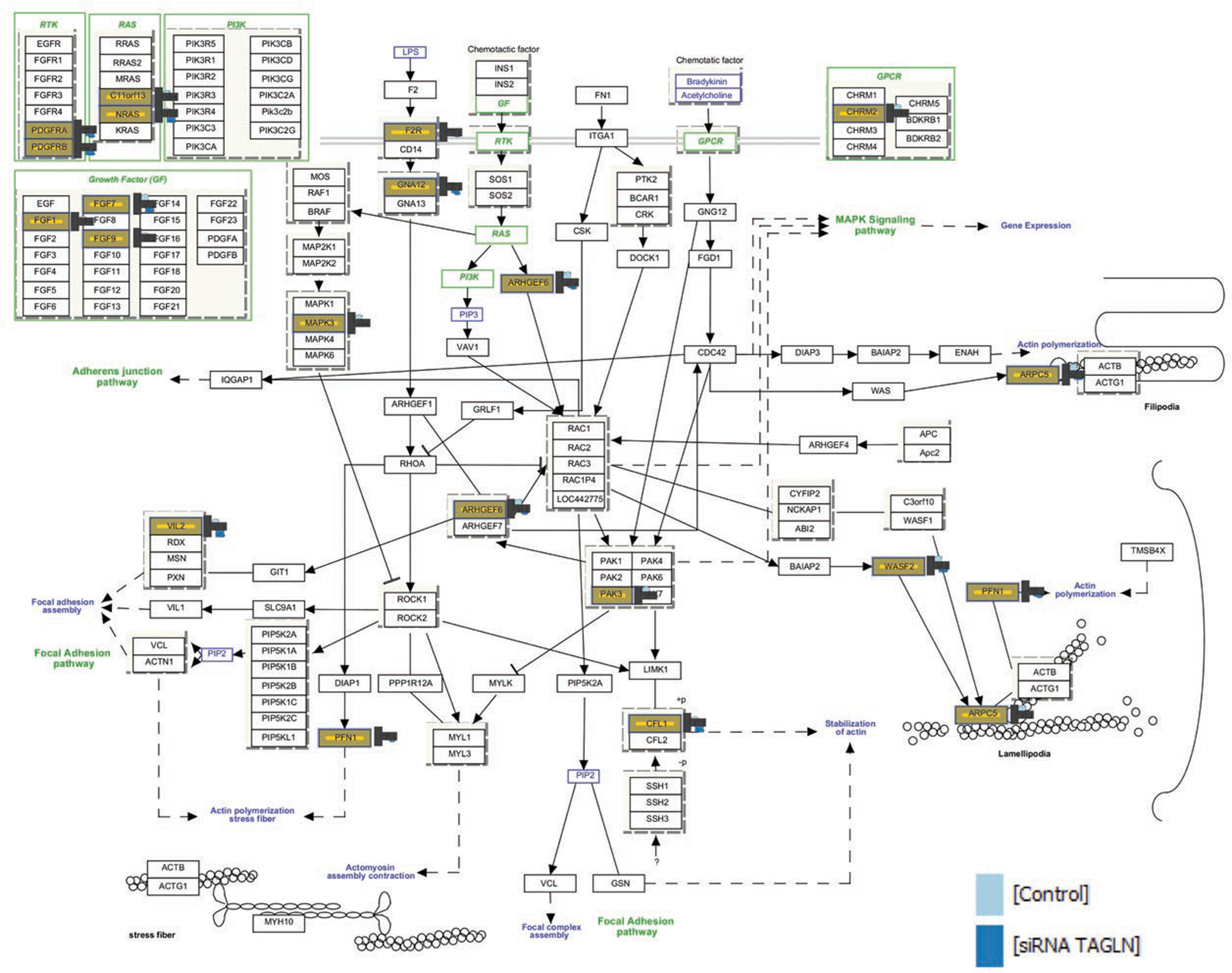

Supplement: Supplementary Figure 8 [file cddis2016196x9.tif]

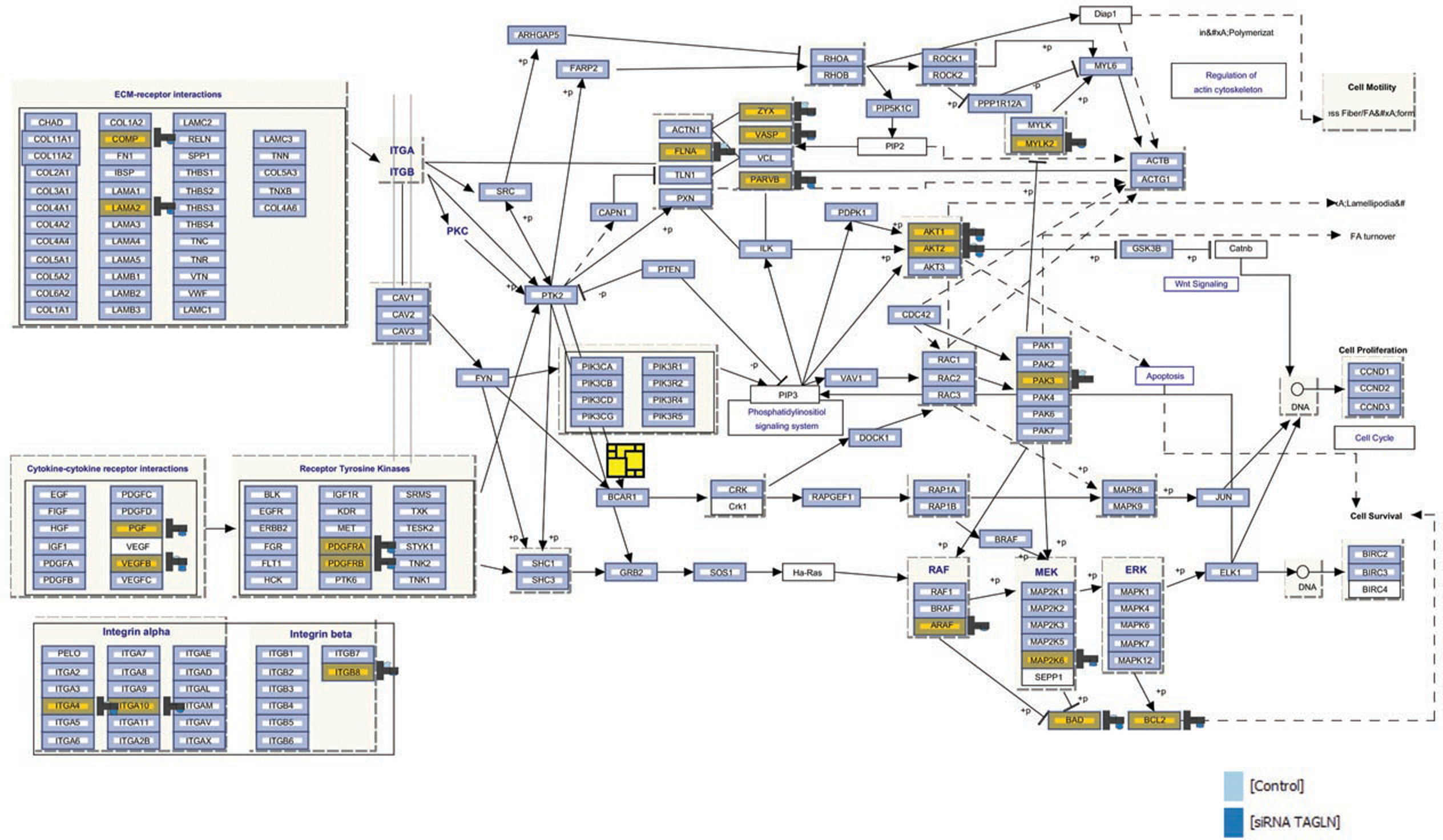

Supplement: Supplementary Figure 9 [file cddis2016196x10.tif]

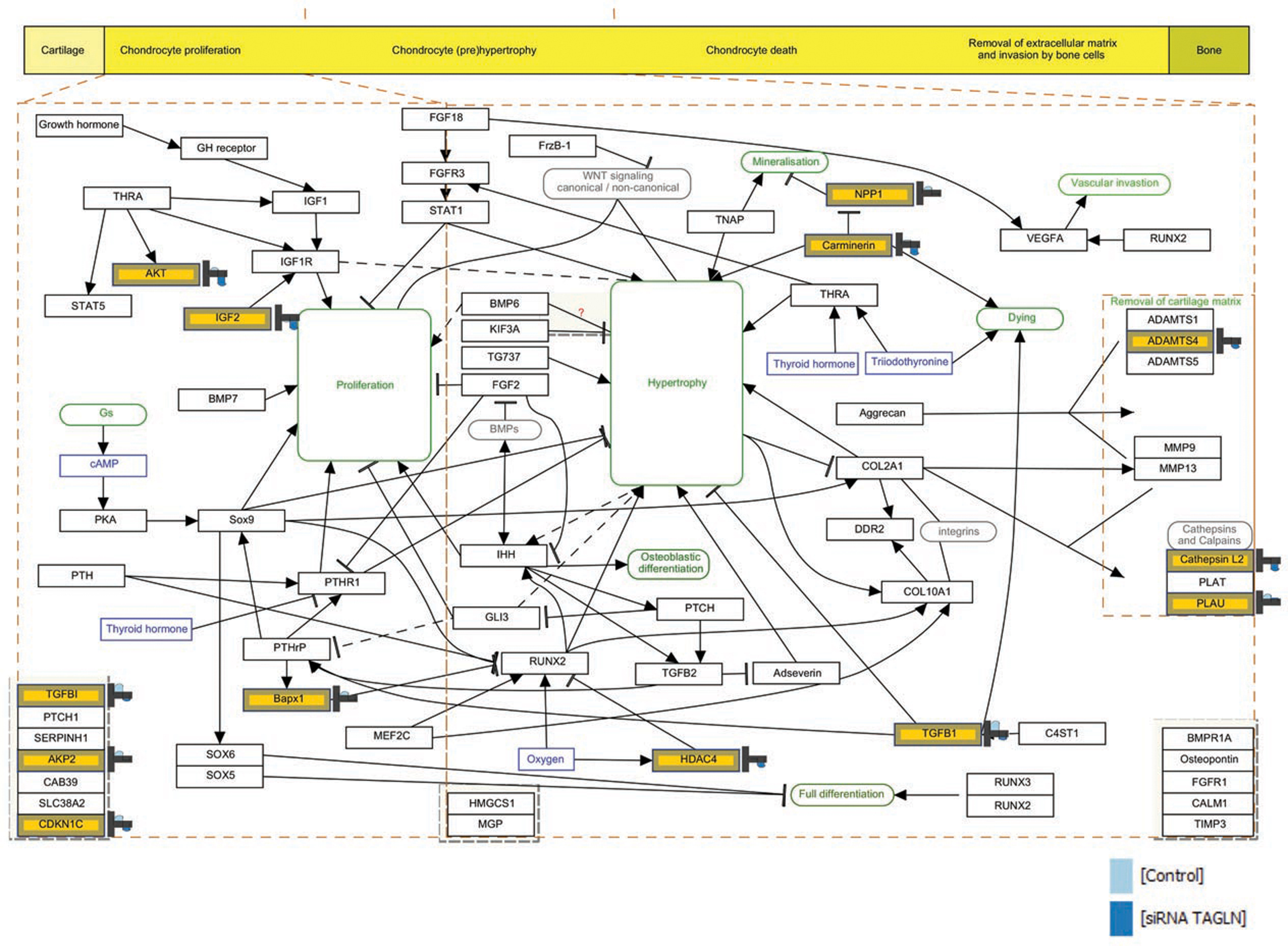

Supplement: Supplementary Figure 10 [file cddis2016196x11.tif]

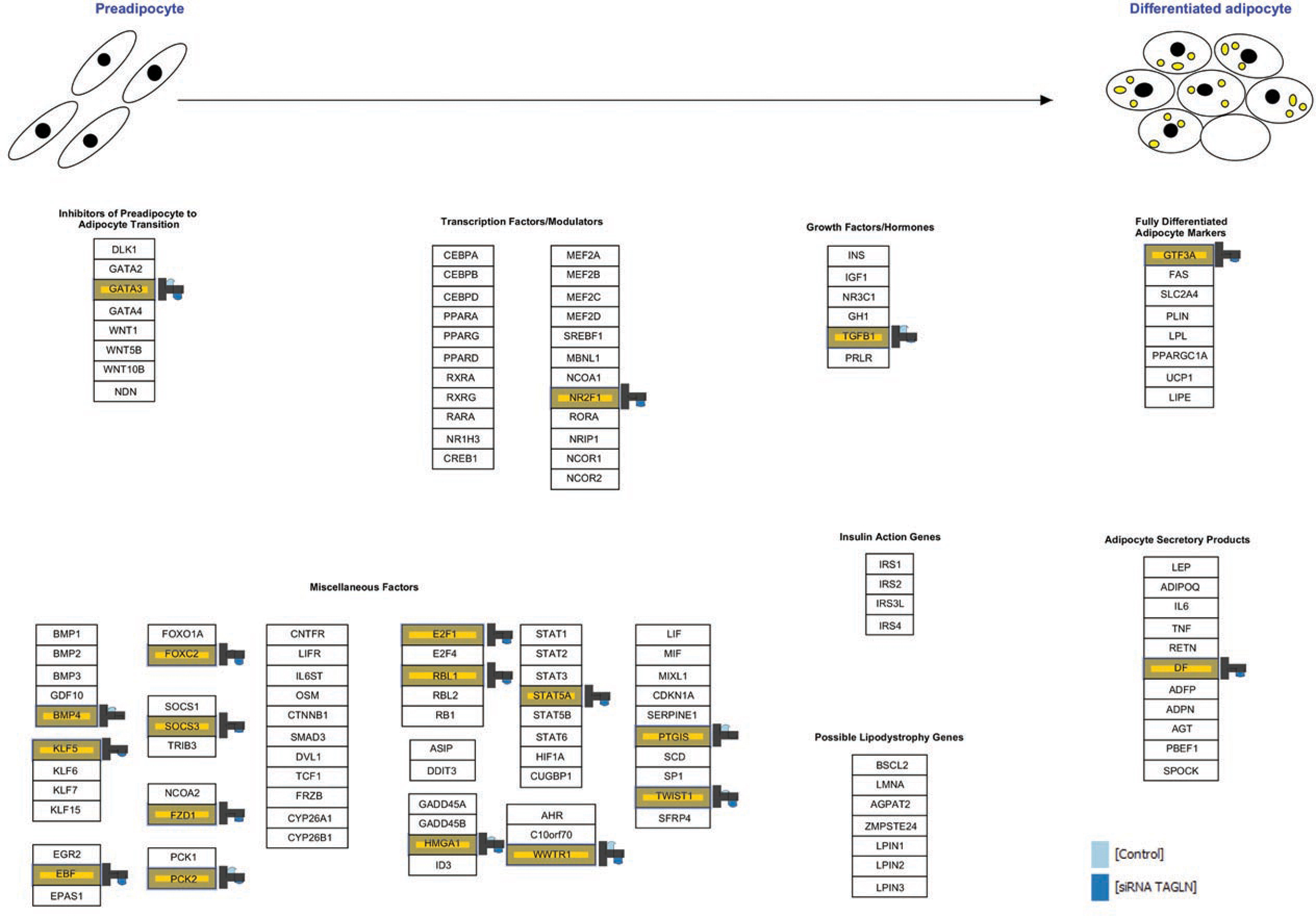

Supplement: Supplementary Figure 11 [file cddis2016196x12.tif]

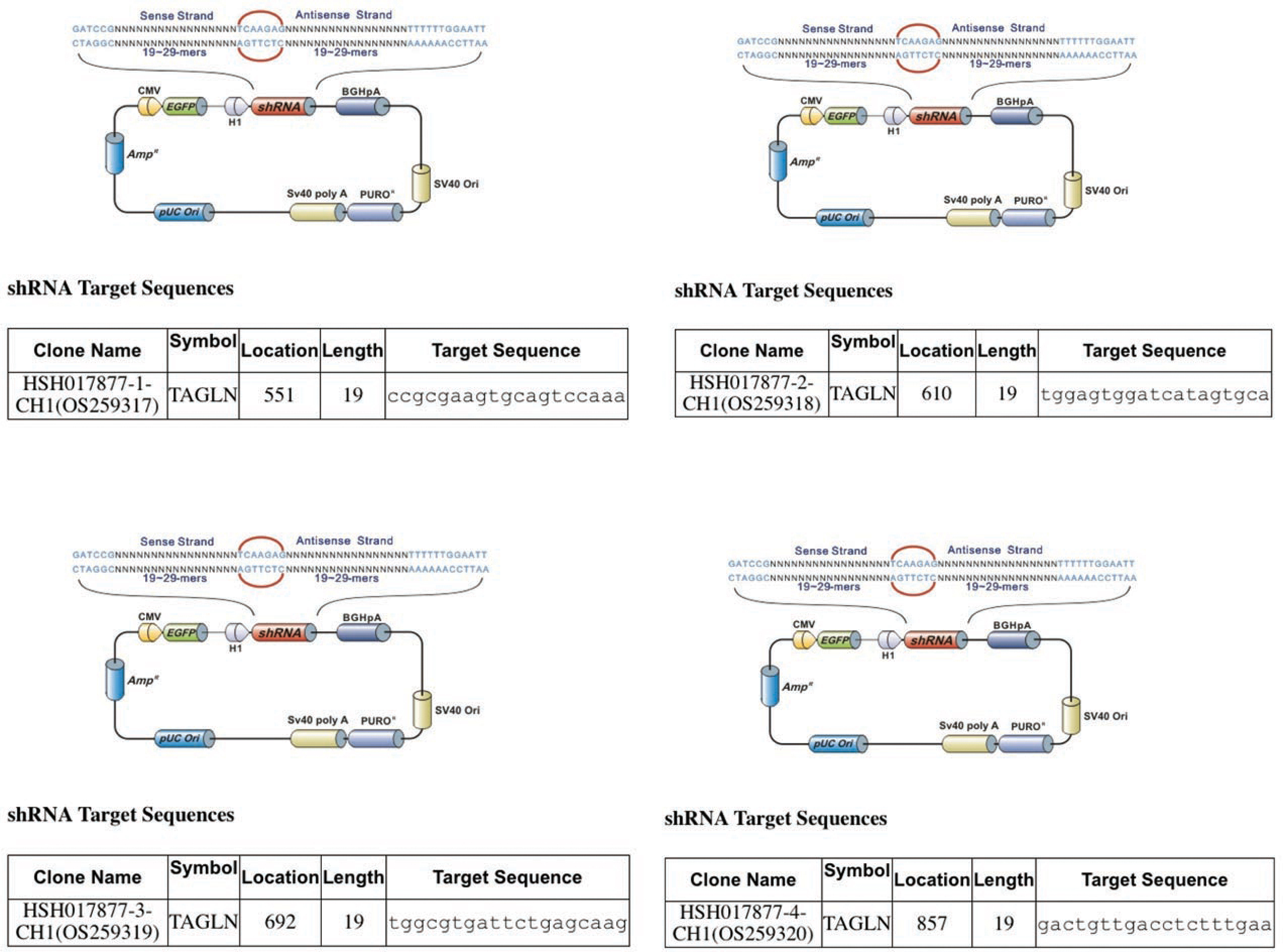

Supplement: Supplementary Figure 12 [file cddis2016196x13.tif]

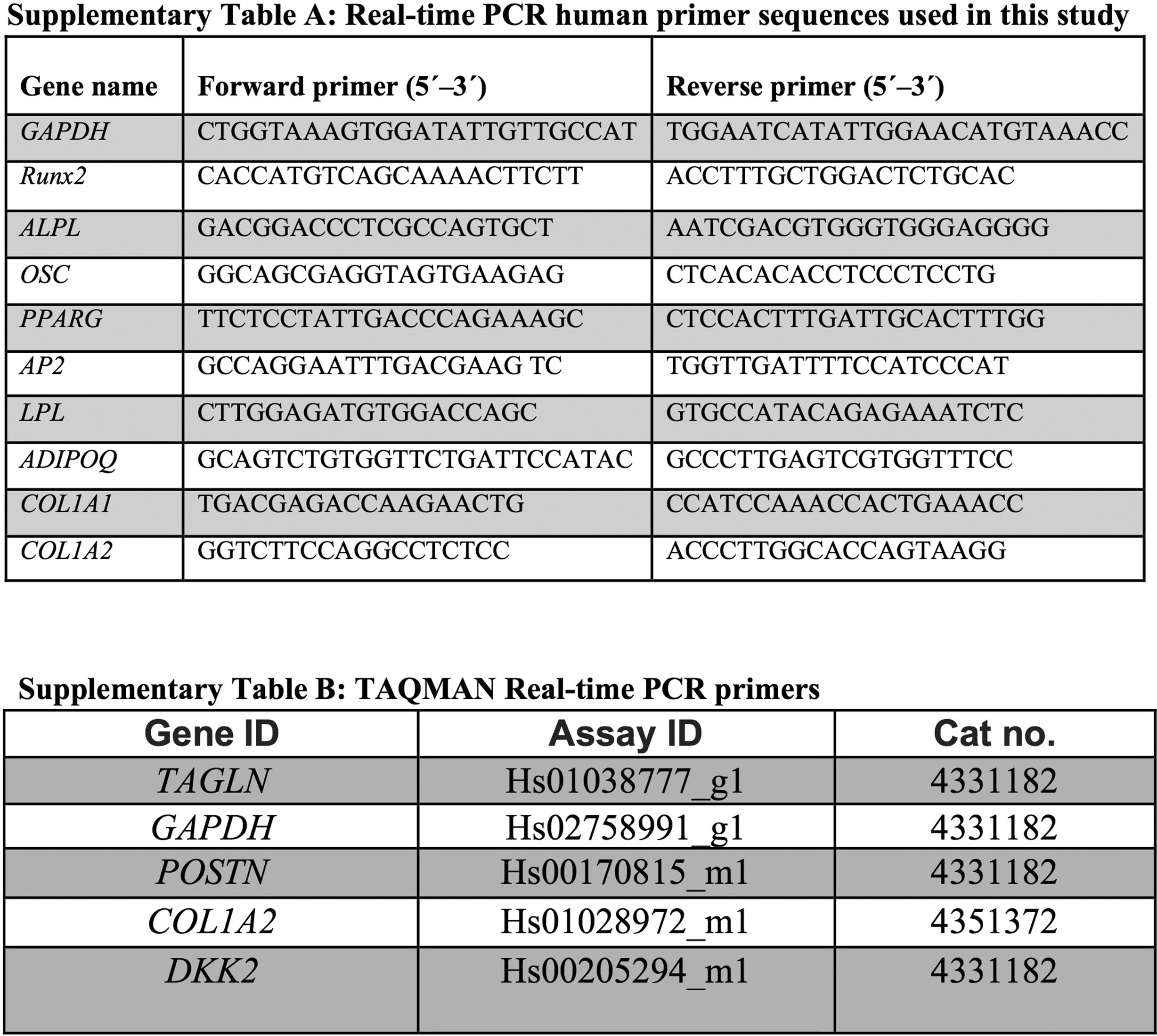

Supplement: Supplementary Table a [file cddis2016196x14.tif]

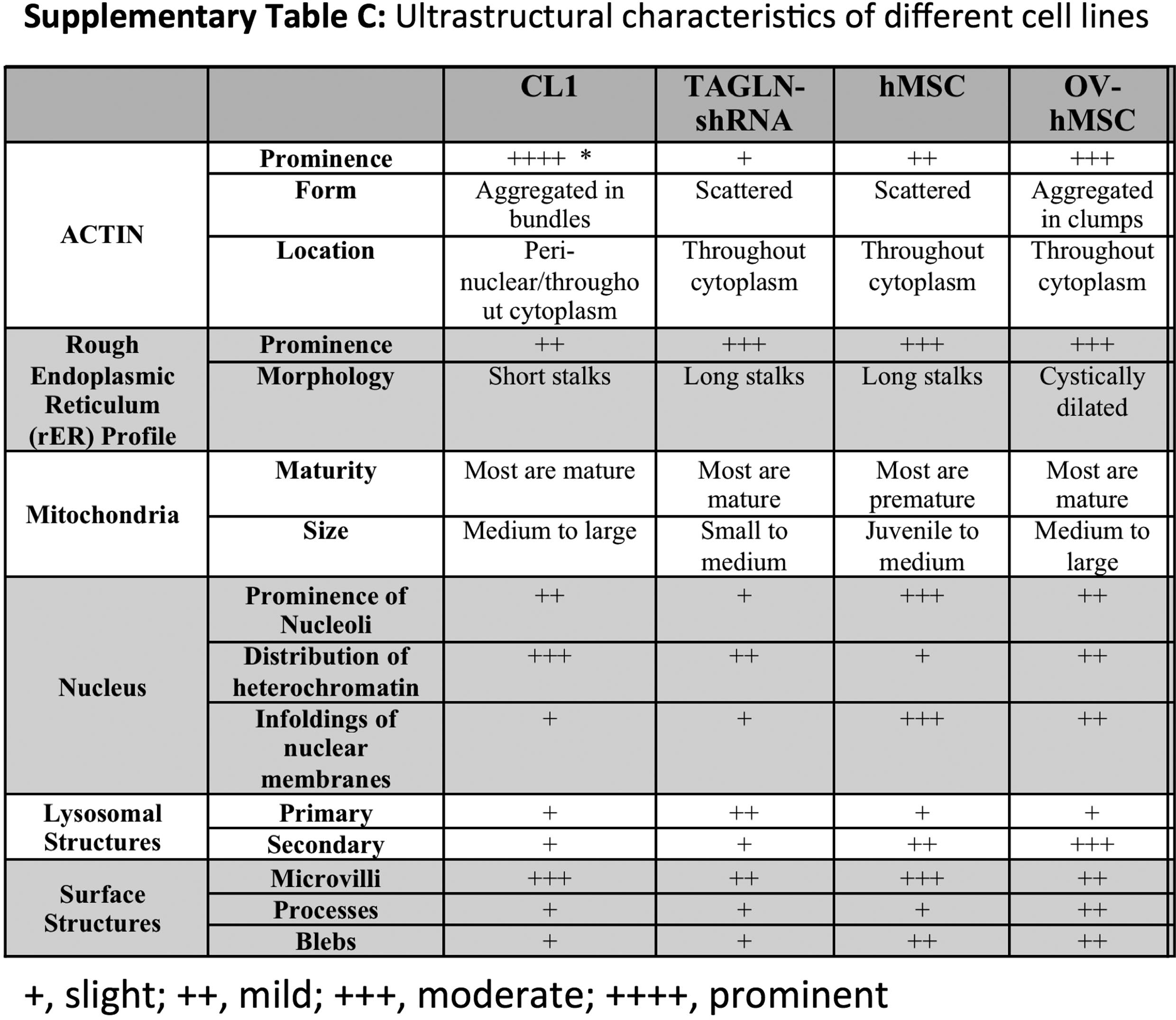

Supplement: Supplementary Table b [file cddis2016196x15.tif]

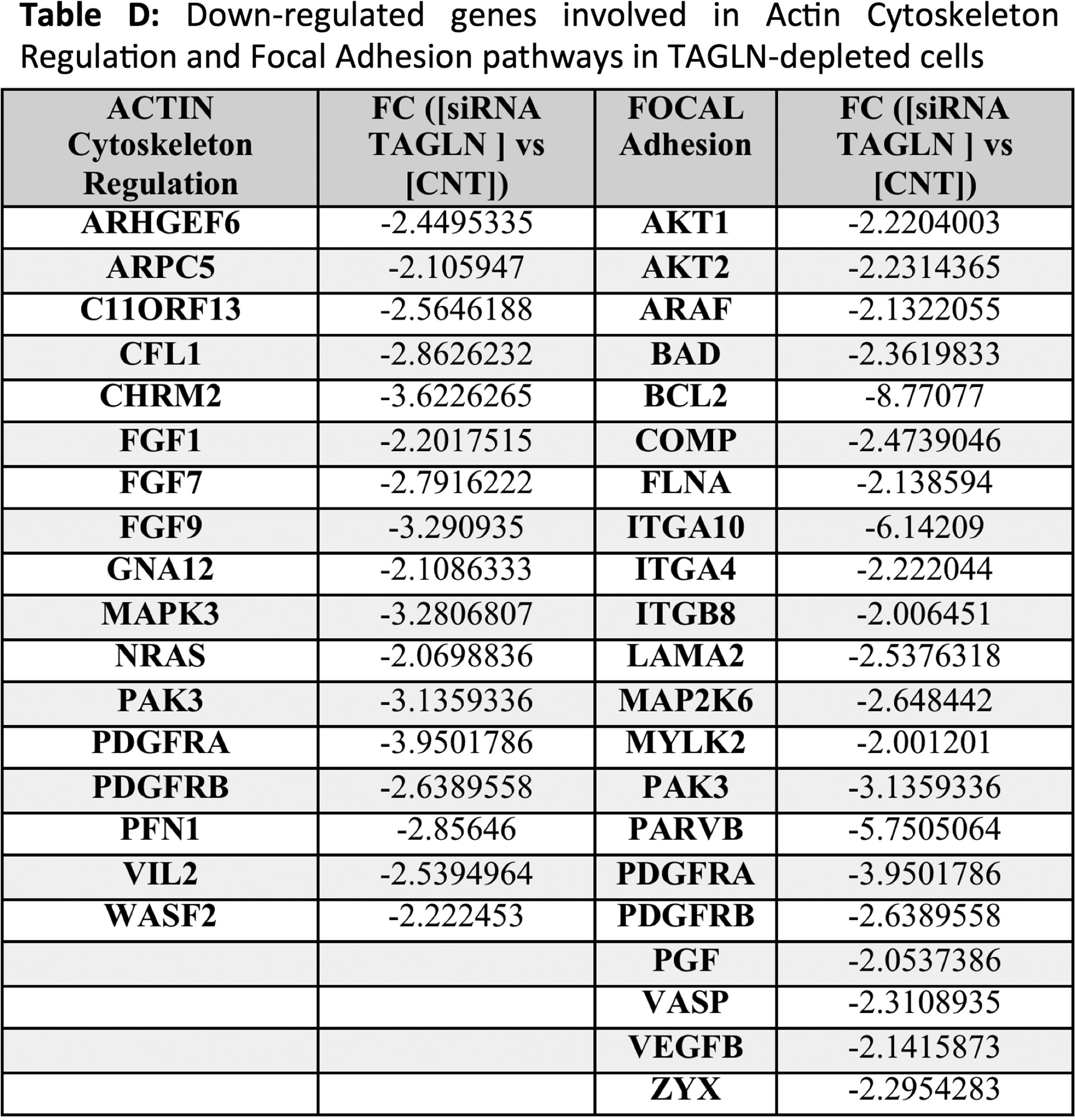

Supplement: Supplementary Table c [file cddis2016196x16.tif]

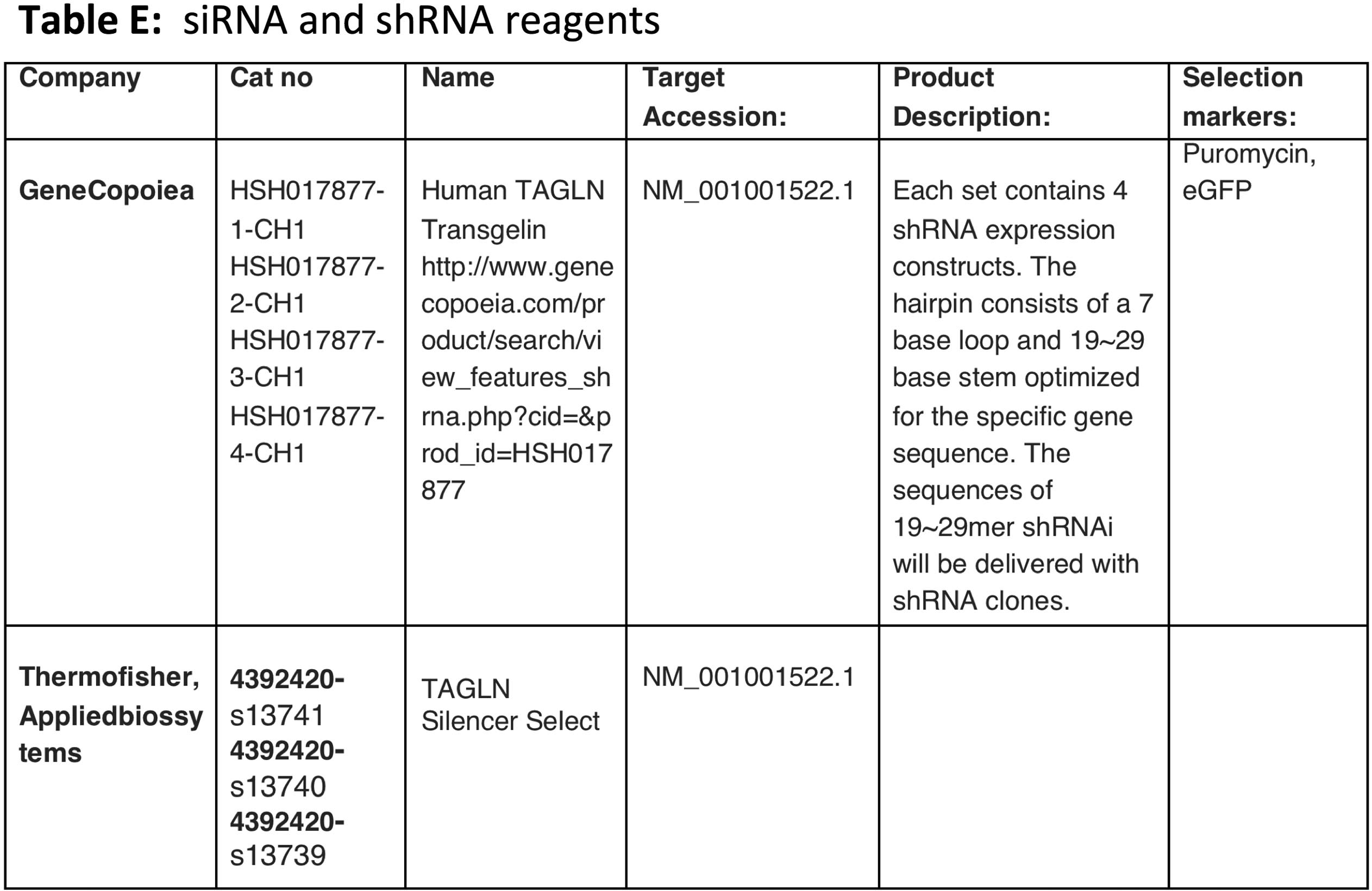

Supplement: Supplementary Table d [file cddis2016196x17.tif]
